# Characterization of SARS-CoV-2 Distribution and Microbial Succession in a Clinical Microbiology Testing Facility during the SARS-CoV-2 Pandemic

**DOI:** 10.1128/spectrum.04509-22

**Published:** 2023-03-14

**Authors:** Govind Prasad Sah, Grace Kovalick, Jessica Chopyk, Peiting Kuo, Lina Huang, Pooja Ghatbale, Promi Das, Susan Realegeno, Rob Knight, Jack A. Gilbert, David T. Pride

**Affiliations:** a Department of Pathology, University of California San Diego, San Diego, California, USA; b Department of Medicine, University of California San Diego, San Diego, California, USA; c Department of Pediatrics, University of California San Diego, San Diego, California, USA; d Center for Microbiome Innovation, University of California San Diego, San Diego, California, USA; e Scripps Institution of Oceanography and Department of Pediatrics, University of California San Diego, San Diego, California, USA; f Department of Bioengineering, University of California San Diego, San Diego, California, USA; g Department of Computer Science & Engineering, University of California San Diego, San Diego, California, USA; David Geffen School of Medicine at UCLA

**Keywords:** built environment, SARS-CoV-2, RT-qPCR, microbiota, clinical microbiology lab

## Abstract

The exchange of microbes between humans and the built environment is a dynamic process that has significant impact on health. Most studies exploring the microbiome of the built environment have been predicated on improving our understanding of pathogen emergence, persistence, and transmission. Previous studies have demonstrated that SARS-CoV-2 presence significantly correlates with the proportional abundance of specific bacteria on surfaces in the built environment. However, in these studies, SARS-CoV-2 originated from infected patients. Here, we perform a similar assessment for a clinical microbiology lab while staff were handling SARS-CoV-2 infected samples. The goal of this study was to understand the distribution and dynamics of microbial population on various surfaces within different sections of a clinical microbiology lab during a short period of 2020 Coronavirus disease (COVID-19) pandemic. We sampled floors, benches, and sinks in 3 sections (bacteriology, molecular microbiology, and COVID) of an active clinical microbiology lab over a 3-month period. Although floor samples harbored SARS-CoV-2, it was rarely identified on other surfaces, and bacterial diversity was significantly greater on floors than sinks and benches. The floors were primarily colonized by bacteria common to natural environments (e.g., soils), and benchtops harbored a greater proportion of human-associated microbes, including Staphylococcus and Streptococcus. Finally, we show that the microbial composition of these surfaces did not change over time and remained stable. Despite finding viruses on the floors, no lab-acquired infections were reported during the study period, which suggests that lab safety protocols and sanitation practices were sufficient to prevent pathogen exposures.

**IMPORTANCE** For decades, diagnostic clinical laboratories have been an integral part of the health care systems that perform diagnostic tests on patient’s specimens in bulk on a regular basis. Understanding their microbiota should assist in designing and implementing disinfection, and cleaning regime in more effective way. To our knowledge, there is a lack of information on the composition and dynamics of microbiota in the clinical laboratory environments, and, through this study, we have tried to fill that gap. This study has wider implications as understanding the makeup of microbes on various surfaces within clinical laboratories could help identify any pathogenic bacterial taxa that could have colonized these surfaces, and might act as a potential source of laboratory-acquired infections. Mapping the microbial community within these built environments may also be critical in assessing the reliability of laboratory safety and sanitation practices to lower any potential risk of exposures to health care workers.

## INTRODUCTION

Clinical laboratories routinely conduct a variety of diagnostic tests to provide reliable data to assist physicians in diagnosing diseases in their patients. Due to the nature of the service provided, clinical laboratories usually are near hospitals or housed in a separate section of the same building. According to the United States Department of Labor, an estimated 500,000 workers are employed in clinical laboratories across the USA. These workers, particularly those working in microbiology sections, are at greater risk of infections caused by a wide variety of microorganisms that fall under the category of occupational hazards. Laboratory-acquired infections (LAIs) are defined as infections acquired through laboratory-related activities (1), and could potentially be symptomatic depending on the microbe acquired. According to a survey conducted by doctoral-level clinical microbiology laboratory directors in 2005, 33% of laboratories reported at least one LAI over the period of 3 years from 2002 to 2004 (2). These employees may acquire LAIs through improper use of personal protective equipment, contaminated work surfaces, or a lack of adherence to safety protocols. There have been several outbreaks of Salmonella over the last decade in microbiology laboratories despite their rigorous safety protocols (3–5). Nevertheless, LAIs have been decreasing in recent years, likely due to improved ventilation, process changes, and greater adherence to training and safety protocols (6, 7). Some of the most concerning causative agents of LAIs include Brucella sp., *Shigella* sp., Salmonella sp., Mycobacterium tuberculosis, and Neisseria meningitidis (8).

Contaminated surfaces can act as sources of pathogen transmissions between individuals (9) and can substantially impact human health. Viruses are frequently transmitted, particularly in indoor environments; depending on the virus and the host, symptoms may range from none to severe life-threatening conditions (10). Most viruses causing respiratory tract infections (e.g., coronavirus, coxsackie virus, influenza virus, respiratory syncytial virus, and rhinovirus) can persist on surfaces for days, and disseminate infection if the surfaces are not properly disinfected (11). Coronavirus Induced Disease 19 (COVID-19) is caused by the Severe Acute Respiratory Syndrome Coronavirus type 2 (SARS-CoV-2), which has been responsible for 520 million cases and 6.2 million deaths worldwide between January 2020 and April 2022 (12). The primary mode of SARS-CoV-2 transmission is respiratory droplets released into the air by coughing, sneezing, speaking, and singing (13–16). Toward the beginning of the pandemic, some early laboratory studies revealed the persistence of SARS-CoV-2 virus on human skin, plastic, glass, cloth, stainless steel, and other surfaces for hours to days (17–19). A plethora of research has been done on the prevalence of SARS-CoV-2 virus and RNA was found on every conceivable object/surface, which suggested the possibility of fomite borne viral dissemination (20–23). In some studies of viral persistence in controlled laboratory environments, and viral detection in real-world settings, majority of swab samples showed positive PCR tests; however only a handful showed limited cytopathic effect (24–30), suggesting that the virus was not viable on surfaces, and, therefore, illustrates that SARS-CoV-2 surface transmission may be a possibility but not a rule (31).

Microbes are ubiquitously present in nature, and their distribution, diversity, and dispersal are shaped by their ability to adapt and compete within their surrounding environment. Modern humans spend approximately 90% of their time inside the built environment (BE) (32–34), and the microbiology of these environments are primarily shaped by the microbial profiles of the individuals inhabiting them (35). The microbes that inhabit the BE are probably most important in health care settings, where chronically ill patients are at high risk of acquiring hospital-acquired infections. These types of infections are among the leading causes of patient deaths (36–39); however, there have been relatively few studies characterizing the microbes that exist in health care environments (40–51). Prior studies have demonstrated that both pathogen outbreaks and pathogen exposures can occur in a clinical microbiology laboratory setting, because the people in the laboratory work to cultivate and/or detect these pathogens. Interestingly, there have been no prior reports detailing the microbiology of surfaces within the clinical microbiology laboratory to help determine whether these surfaces could harbor potential pathogens.

There has been a rapid growth in the discovery and application of culture-independent techniques, such as high-throughput DNA sequencing that has greatly increased our understanding of the complex microbial communities that inhabit the BE (52, 53). Specifically, molecular investigations using the 16S rRNA marker gene have enabled the identification of novel, previously uncultivable bacterial species under normal laboratory conditions (54). In recent years, 16S rRNA amplicon sequencing has facilitated the study of microbes inhabiting a variety of BEs (55, 56). The hospital microbiome is primarily shaped by patients and health workers, and may be more diverse and dynamic compared to other BEs (50, 57). Likewise, studies focusing on intensive care units (ICUs) have reported increased abundances of skin-associated microbes (49), but with reduced diversity compared to nonpatient care portions of the hospital (41, 50, 58). We recently characterized the microbial composition of different surfaces in the ICU during different stages of a renovation (45). Our results demonstrated that microbial composition is significantly influenced by environmental and humans-associated bacteria at each stage of renovation.

Clinical laboratories have been operating and performing diagnostic tests for decades, and the makeup of microbes on their surfaces have not been thoroughly examined. When working surfaces are contaminated with pathogens, they may serve as an indirect source of disease transmission. Therefore, identifying pathogens that potentially inhabit these surfaces is of critical importance. Such information may also be critical in assessing the reliability of laboratory safety and sanitation practices to lower any potential risk of exposures. During times where laboratories are working with novel pathogens, such as with the arrival of the SARS-CoV-2 pandemic, such studies to characterize where these pathogens may reside within the clinical laboratory may help to elucidate exposure risks and inform sanitation practices. Here, we examined the surfaces of a clinical microbiology facility to determine whether surfaces within the laboratory may present potential exposure risks, and to identify whether succession of microbes in the laboratory is human-associated. We chose several different parts of the laboratory, including a basic bacterial culture section, a molecular microbiology section, and a SARS-CoV-2 testing section to identify microbiological trends that may be informative. We analyzed bacterial succession longitudinally using 16S rRNA amplicon sequencing, and tested for the presence of SARS-CoV-2 on laboratory surfaces using reverse transcription quantitative polymerase chain reaction (RT-qPCR).

## RESULTS

### SARS-CoV-2 testing.

**FIG 1:**
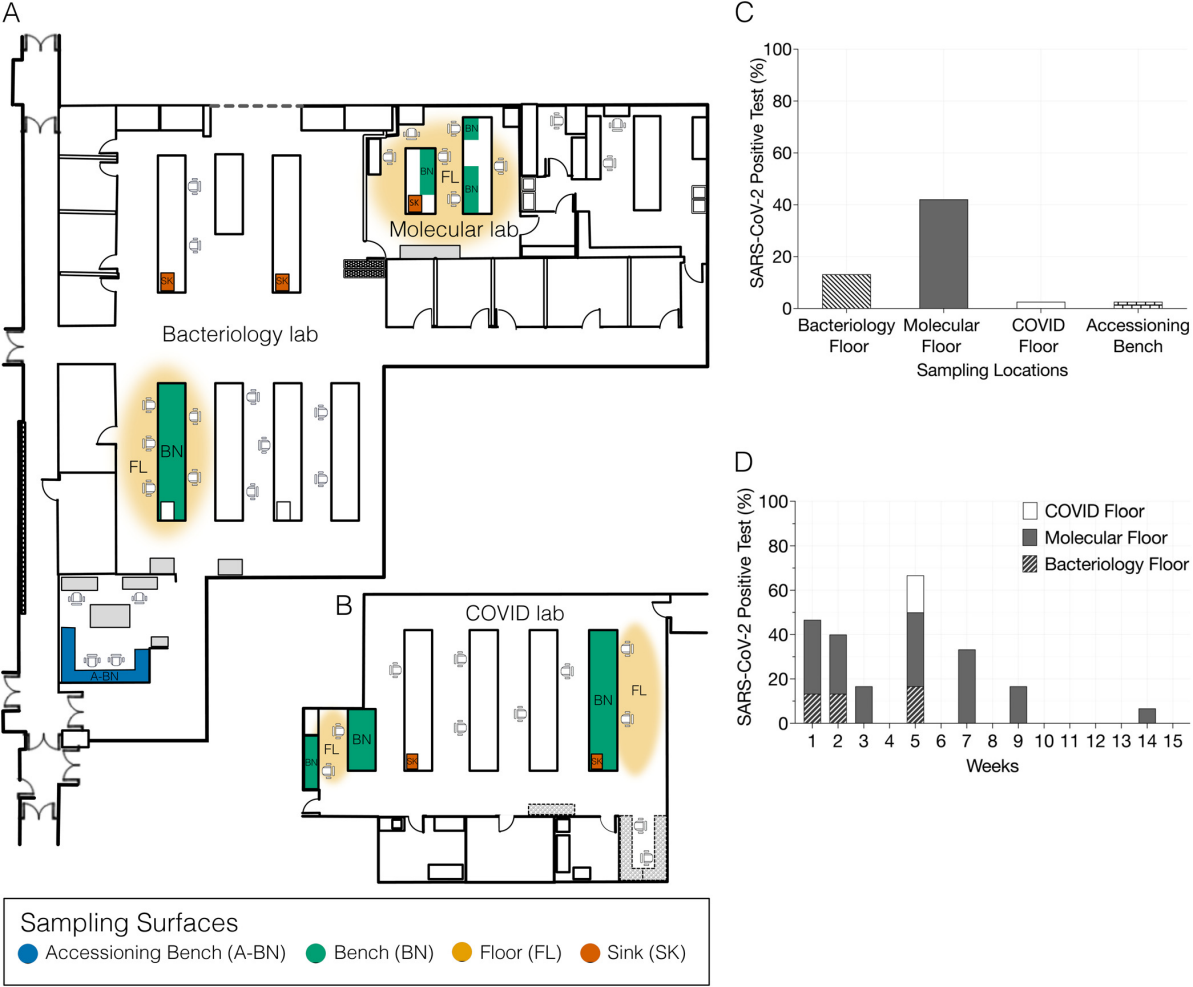
Representative map of clinical lab sections highlighting sampling surfaces and SARS-CoV-2 test results. Map of clinical bacteriology, molecular (A) and COVID testing lab (B) showing the sampling surfaces; Accessioning bench (A-BN), Bench (BN), Floor (FL) and Sink (SK). COVID testing lab is physically separated from the other 2 lab sections and is a relatively newer setup. Maps are not to scale. Summary of the quantification of SARS-CoV-2 RT-qPCR testing of the clinical lab sections grouped by surface (C) and by time (D).

The COVID overflow section did not officially open until 10/13/2020, and was used primarily as a control for a laboratory that had very little influence from people working on its premises. The bacteriology section, where bacterial cultures are performed routinely, also included the main A-BN, which is the entry point for all the specimens into the laboratory. A separate part of the main bacteriology section was devoted to serological testing for antibodies and antigens. Diagnostic tests in the molecular microbiology section included work related to detection of DNA, RNA, proteins, and other small molecules from patient samples using molecular techniques, such as PCR and hybridization. Even though the laboratory developed a separate space for some of the SARS-CoV-2 diagnostics (COVID overflow lab section), most of the SARS-CoV-2 testing occurred in the molecular microbiology section throughout the study. We used the TaqPath COVID-19 (Thermo Fisher Scientific) quantitative PCR test for SARS-CoV-2 detection from each of the swab specimens. This assay evaluates the presence of 3 gene targets from the orf1a/b, S, and N regions of the SARS-CoV-2 genome to determine their presence/absence in a sample. At least 2 of the 3 genes must be amplified for any specimen to be called positive. We found that the floor (FL) in the molecular microbiology laboratory had the greatest presence of SARS-CoV-2 of all the surfaces tested, with 16 out of 38 samples tested positive (42.10%). Similarly, the floor samples from bacteriology overflow laboratory had 13.2% (5 out of 38 samples positive) and the COVID overflow lab had 2.6% (1 out of 38 samples positive) of samples tested positive, respectively (Fig. 1C and Table 1). We only identified a single positive swab specimen taken from the accessioning bench out of 38 tested (2.6%). Of note, the A-BN is the access point where all the specimens are received and enter the laboratory. Swab specimens from other surfaces within each of the laboratory sections were found to be free of virus, including the other benches (BN) and sinks (SK).

**TABLE 1:** SARS-CoV-2 positive RT-PCR test showing Ct value for the three genes

				Ct value		
SN^*a*^	Sample ID	Date	Surface	ORF1a/b gene	S gene^*b*^	N gene
1	6	7/20/20	Molecular Floor	31.4	33.2	30.6
2	13	7/21/20	Bacteriology Floor	34.2	-	33.2
3	16	7/21/20	Molecular Floor	27.4	29.2	26.6
4	26	7/22/20	Molecular Floor	32.2	30.8	31.5
5	33	7/23/20	Bacteriology Floor	31.0	32.7	29.0
6	36	7/23/20	Molecular Floor	27.6	29.6	27.0
7	46	7/24/20	Molecular Floor	35.0	-	33.2
8	56	7/27/20	Molecular Floor	30.8	32.9	29.8
9	63	7/28/20	Bacteriology Floor	33.6	-	32.5
10	66	7/28/20	Molecular Floor	34.3	-	33.0
11	76	7/29/20	Molecular Floor	31.5	33.3	30.2
12	83	7/30/20	Bacteriology Floor	34.0	-	32.4
13	86	7/30/20	Molecular Floor	36.0	-	32.9
14	91	7/31/20	Accessioning Bench	33.9	-	33.0
15	106	8/3/20	Molecular Floor	33.9	29.2	34.5
16	143	8/17/20	Bacteriology Floor	35.7	-	32.9
17	146	8/17/20	Molecular Floor	32.2	23.1	31.5
18	149	8/17/20	COVID Floor	30.5	24.8	30.6
19	156	8/21/20	Molecular Floor	32.5	35.7	32.4
20	186	8/31/20	Molecular Floor	30.7	36.8	31.0
21	196	9/4/20	Molecular Floor	33.5	37.0	34.6
22	226	9/14/20	Molecular Floor	33.9	36.0	34.1
23	316	10/22/20	Molecular Floor	33.8	-	36.6

a SN, Serial Number.

b -, S-gene was not detected in some samples.

Next, we performed a longitudinal analysis to evaluate the prevalence of SARS-CoV-2 on the laboratory floor surfaces over the entire 15-weeks of the study period. In the first week, we confirmed 46.67% of all the floor swab samples tested to be positive (7 of 15 samples) which declined to 40% and 16.67% in the 2nd and 3rd week respectively. The cases declined over the weeks with an unexpected hike to 66.67% (4 of 6 samples) in week 5 (Fig. 1D). We found that the majority of SARS-CoV-2 positive floor samples were from the molecular lab section, which highlights the role of molecular diagnostic technique as a potential source of viral dispersal and contamination in laboratory settings.

### Alpha diversity among surfaces differed significantly.

**FIG 2:**
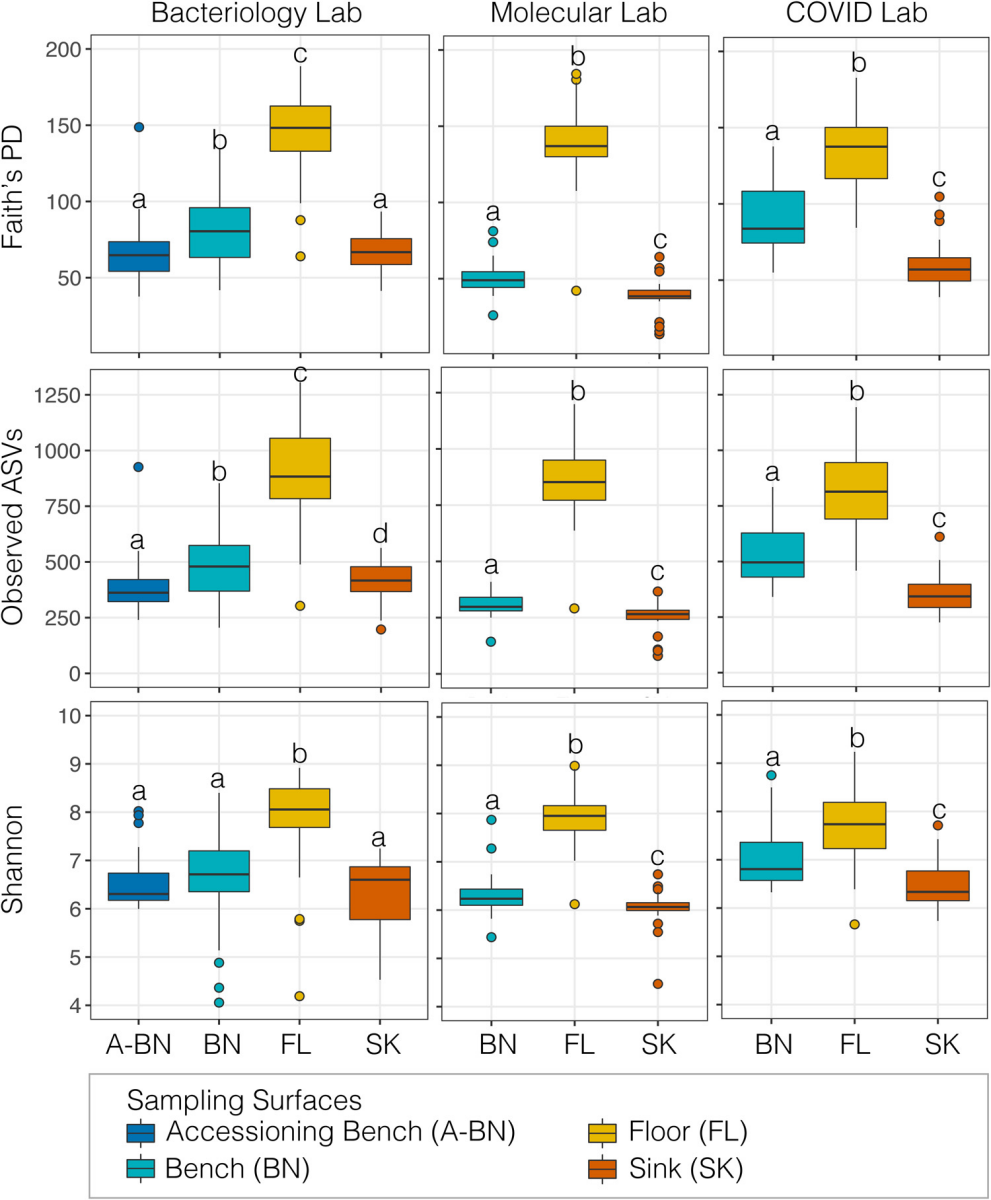
Alpha diversity boxplot showing Faith's PD (top panel), Observed ASVs (middle panel) and Shannon's index (bottom panel) for bacteriology, molecular and COVID overflow lab sections with samples grouped by sampling surfaces. Alpha diversity metrics (Faith's PD, Observed ASVs, and Shannon) are shown on the *y* axis in different panels, while sampling surface groups are shown on the *x* axis. Letters (“a,” “b,” “c, and” “d”) shared in common among the different surfaces for each laboratory denotes no significant difference (p_adj_ > 0.05) as determined by the Kruskal-Wallis test. For example, Shannon index did not differ among A-BN, BN, and SK of the Bacteriology lab section while FL differed significantly from others. The boxplots show the Interquartile Range (IQR) between the first and third quartiles, the center line is the median, where Whiskers represent the smallest (y-min) and largest (y-max) observations within 1.5 times the IQR from the first and third quartiles, respectively. Outliers are indicated by respective colored circles as shown in the legend.

In each laboratory section (bacteriology, molecular, and COVID) and sampling surfaces (accessioning benches [A-BN], BN, FL, and SK), we measured alpha diversity of the bacterial communities i.e., the Shannon index (a quantitative measure that accounts for the number of species living in a habitat [richness], their relative abundance [evenness], and Faith’s PD [a qualitative measure of community richness that incorporates phylogenetic differences between species]). Regardless of the metrics used, there were significant differences in the alpha diversity among sampling surfaces for all the lab sections (Fig. 2) as indicated by Kruskal-Wallis test (Faith’s PD; p_adj_ < 0.05). Pairwise comparison between sampling surfaces within individual lab sections indicates that microbial diversity of FL was invariably higher compared to the other surfaces across all the lab sections (Table 2). Likewise, we noticed that the pairwise difference in alpha diversity between all the other surfaces was significant (Table 2), except between A-BN and SK within the Bacteriology Lab section (H = 1.30, p_adj_ = 0.253). Surprisingly, we noted a significant difference in the alpha diversity between A-BN and BN surfaces (Fig. 2 and Table 2) (H = 9.135, p_adj_ < 0.05). This could be due to the fact that, although both A-BN and BN represent benches, the BN in the other sections of the lab represent surfaces where microbial cultivation and PCR takes place, while the A-BN simply represents an access point for specimens into the clinical microbiology lab. Furthermore, to test the differences in microbial diversity within lab sections, we performed alpha diversity analysis with lab sections as groups, and found that lab sections differed significantly from each other in relation to their benches and sinks but not the floors (Fig. S1). Finally, we studied the longitudinal variation in alpha diversity within individual lab sections, and did not find any significant change over the sampling period (Fig. S2) as indicated by Kruskal-Wallis test (Faith’s PD; p_adj_ > 0.05).

**TABLE 2:** Faith’s PD Kruskal-Wallis (pairwise) test

Group 1	Group 2	H-value	P_adj_^*a*^-value
Bacteriology lab			
Accessioning Bench (*n* = 33)	Bench (*n* = 34)	9.13	3.76 × 10^−03^
Accessioning Bench (*n* = 33)	Floor (*n* = 29)	39.68	8.98 × 10^−10^
Accessioning Bench (*n* = 33)	Sink (*n* = 34)	1.30	2.54 × 10^−01^
Bench (*n* = 34)	Floor (*n* = 29)	36.14	3.66 × 10^−09^
Bench (*n* = 34)	Sink (*n* = 34)	6.32	1.43 × 10^−02^
Floor (*n* = 29)	Sink (*n* = 34)	42.00	5.46 × 10^−10^
Molecular Lab			
Bench (*n* = 34)	Floor (*n* = 32)	43.66	5.86 × 10^−11^
Bench (*n* = 34)	Sink (*n* = 34)	25.04	5.61 × 10^−07^
Floor (*n* = 32)	Sink (*n* = 34)	46.94	2.19 × 10^−11^
COVID Lab			
Bench (*n* = 31)	Floor (*n* = 33)	27.23	1.80 × 10^−07^
Bench (*n* = 31)	Sink (*n* = 35)	30.87	4.12 × 10^−08^
Floor (*n* = 33)	Sink (*n* = 35)	48.83	8.35 × 10^−12^

a p_adj_; adjusted *P* value for multiple testing of sampling surfaces.

### Beta diversity among the clinical laboratory sections.

**FIG 3:**
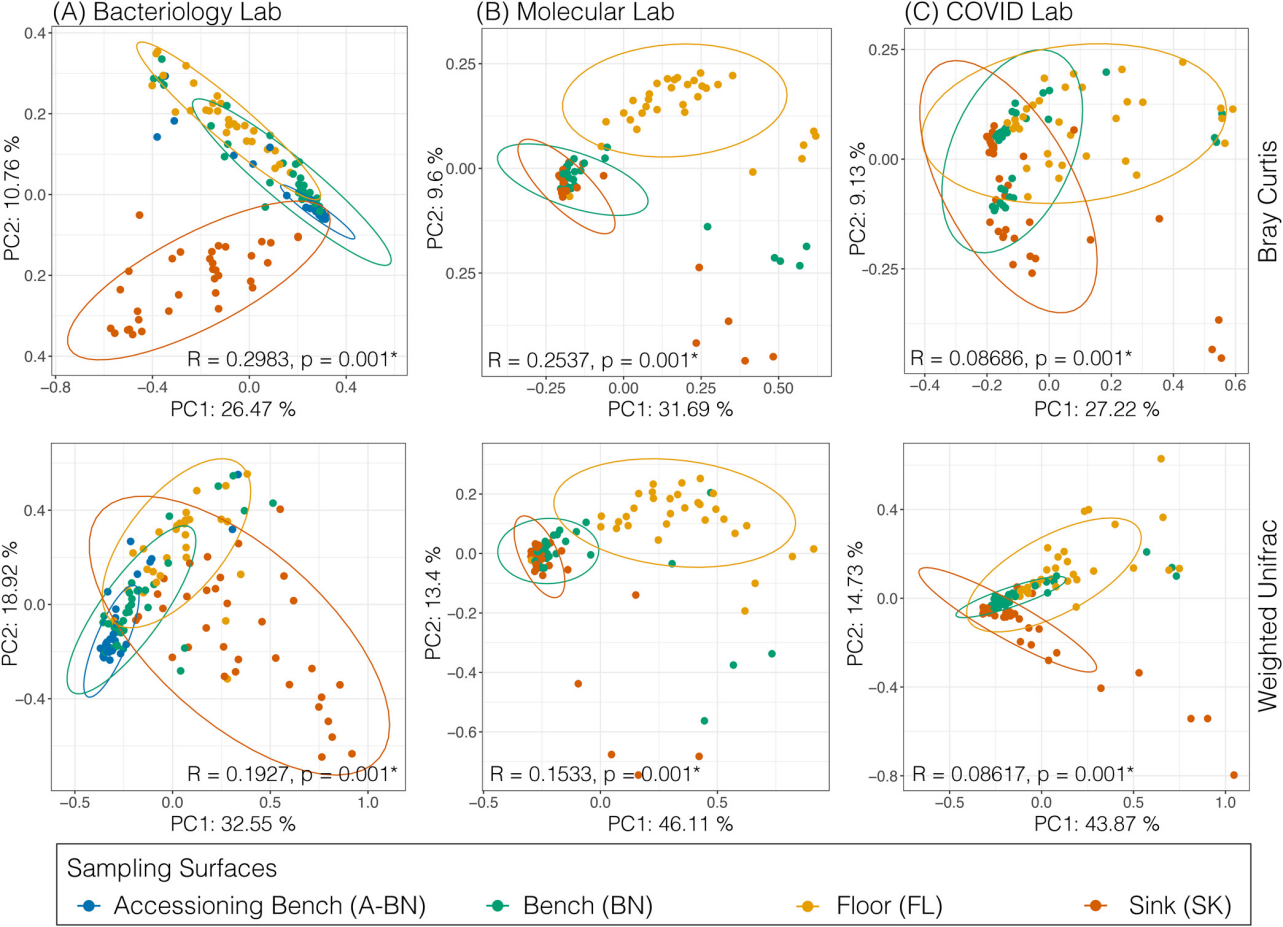
Principal coordinates analysis (PCoA) plot based on Bray Curtis and Weighted Unifrac dissimilarities depicting the clusters of bacterial communities grouped by sampling surfaces for Bacteriology (A), Molecular (B), and COVID (C) lab sections. Ellipses are drawn at 95% confidence intervals for each sampling surface (Accessioning Bench, Bench, Floor, and Sink). Significance determined by ANOSIM with 999 permutations for individual lab sections, and denoted in the corner of each panel *, *P* < 0.05.

Bray Curtis dissimilarity metric showed no significant difference in beta diversity between the lab sections (Fig. S3A) (ANOSIM, *R* = 0.01074, *P* = 0.093). However, the diversity between the sampling surfaces were significantly different as evidenced by separate clustering of Floor and Bench surfaces (Fig. S3B) (ANOSIM; *R* = 0.1196, *P* = 0.001). Next, we stratified the samples by sampling surfaces and lab sections and performed beta diversity analysis using Bray Curtis and Weighted Unifrac metrics. We found that the microbial composition and phylogeny of the sink was very different from floors and benches in the Bacteriology lab (Fig. 3A), and that the floor microbiota was distinct from other surfaces in case of the Molecular and COVID labs (Fig. 3B and C). Furthermore, even though we did not see a clear difference in beta diversity between lab sections when samples are not stratified (Fig. S3A), floors and sinks showed significant difference in beta diversity between lab sections with distinct clustering (Fig. S4). We also examined differences in microbial communities across sampling surfaces from individual lab sections for each sampling day to determine whether microbial communities on surfaces become more or less similar over time. We used Compositional Tensor Factorization (CTF) analysis (59), to examine whether the sampled surfaces within each laboratory section were similar in composition longitudinally. We found that, across the surfaces in each of the different laboratory sections, microbial compositions were highly similar at each time point. For example, there was little variation observed at each time point for the molecular (Fig. S5B) and the COVID (Fig. S5C) sections. Comparatively, we observed a mild degree of compositional difference between different surfaces in the bacteriology section (Fig. S5A), which was not significant.

### Taxonomic profiles and core bacterial taxa by laboratory section.

**FIG 4:**
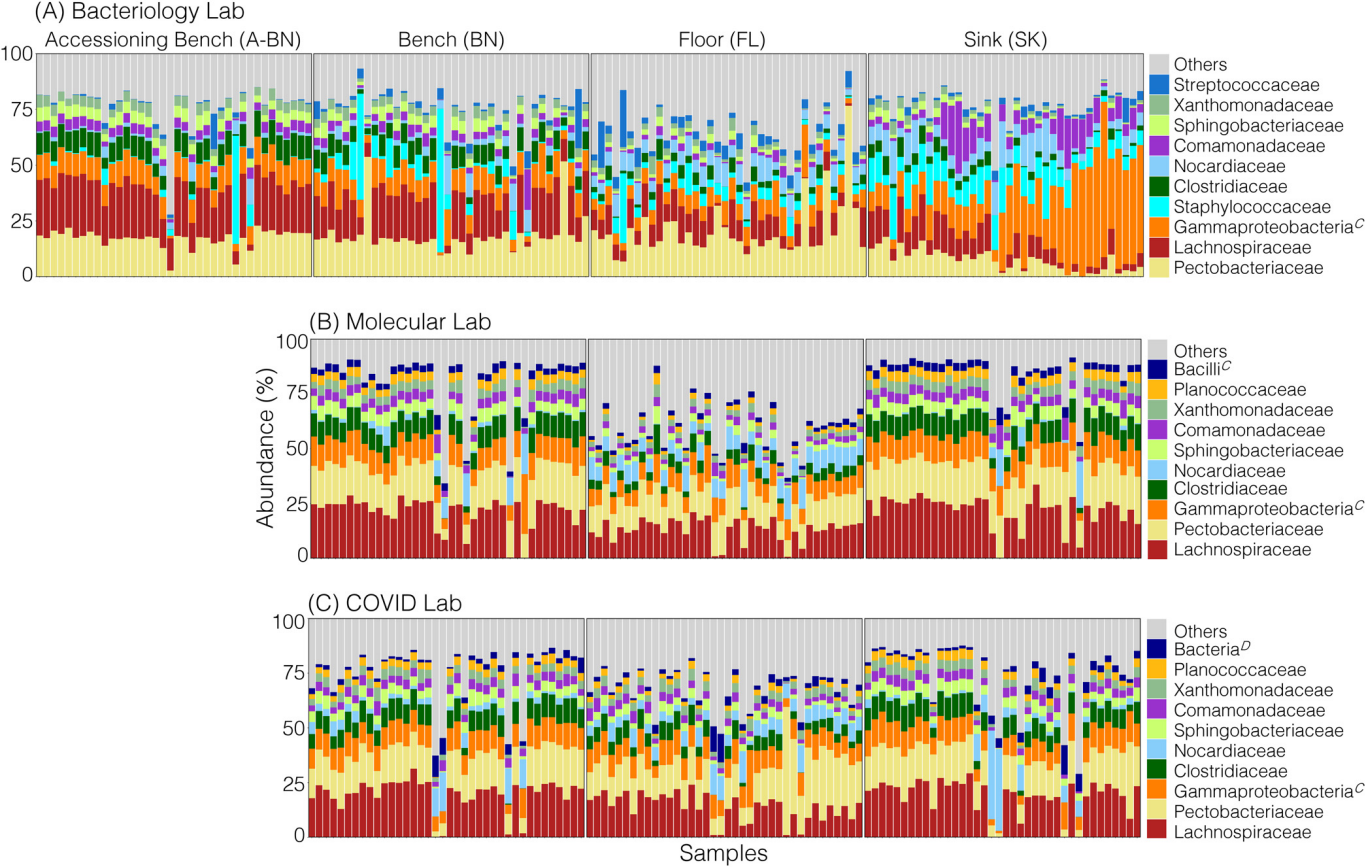
Stacked bar chart of the average relative abundance of the bacterial community composition for samples from bacteriology (A), molecular (B), and COVID (C) overflow lab sections. For each sample source, the percentage relative abundance of the 10 most dominant bacterial families is shown on the *y* axis, and the samples are on the *x* axis. 'Others' comprise the remaining bacterial families in the order of decreasing relative abundance.

Approximately 75% of the microbes we could identify on laboratory surfaces belonged to the families Streptococcaceae, Staphylococcaceae, Lachnospiraceae, Clostridiaceae, Xanthomonadaceae, Nocardiaceae, Comamonadaceae, Sphingobacteriaceae, Pectobacteriaceae, and Planococcaceae (Fig. 4). We identified significantly greater relative abundances of bacteria from families Comamonadaceae, Nocardiaceae, Staphylococcaceae, and class Gammaproteobacteria, while identifying significantly fewer Clostridiaceae, Lachnospiraceae, and Pectobacteriaceae on the sink compared to the benches (Kruskal-Wallis; *P* < 0.05) in the bacteriology lab section (Fig. 4A). Similarly, we saw a significant reduction in the relative abundances of families Clostridiaceae and Lachnospiraceae, while also observing an increase in Nocardiaceae on floor surfaces compared to benches (Kruskal-Wallis; *P* < 0.05) in the bacteriology lab section (Fig. 4A). There was also a significant reduction in the relative abundances of Planococcaceae, Xanthomonadaceae, Comamonadaceae, Sphingobacteriaceae, and Clostridiaceae, accompanied by an increase in the relative abundance of Nocardiaceae on the floor compared to benches and sinks (Kruskal-Wallis; *P* < 0.05) in the molecular section (Fig. 4B). We generally observed less variation in taxonomic profiles on the different surfaces in the COVID section (Fig. 4C) compared to bacteriology (Fig. 4A) and molecular sections (Fig. 4B).

To further understand the core microbial taxa present in different laboratory sections, we computed the “core microbiome,” represented by the taxa present in at least 90% of samples from each group. These taxa belonged to the genera *Dickeya, Comamonas, Sphingobacterium*, families Lachnospiraceae, Xanthomonadaceae, and classes Alphaproteobacteria and Gammaproteobacteria. Actinobacteria was the unique core taxa found on the surfaces of the bacteriology section, while members of the class Alphaproteobacteria were commonly found on the surfaces of all the 3 lab sections. Lachnospiraceae was the only core taxa unique to the molecular lab section.

Next, we evaluated the relative abundance of microbes on different surfaces from these lab sections over the course of the study. These largely included environmental microbes, such as *Rhizobium*, *Nocardia*, Actinobacteria, *Dickeya*, Xanthomonadaceae, and *Sphingobacterium*, but also human-associated microbes including Staphylococcus, and Streptococcus (Fig. S6). There were also microbes, such as gamma-proteobacteria, Lactobacillales, and Lachnospiraceae, which could be derived from humans or from other environmental sources. Many of the taxa identified had relatively stable relative abundances over time, regardless of the surface being examined. Certain microbes, such as *Dickeya*, *Clostridium*, Lachnospiraceae, and gamma-proteobacteria, were relatively common to all surfaces in each of the laboratories. However, Staphylococcus and Streptococcus, which were common to the bacteriology section (Fig. S6A), were less common in the molecular (Fig. S6B) and COVID (Fig. S6C) laboratories.

### Differentially abundant bacterial taxa.

**FIG 5:**
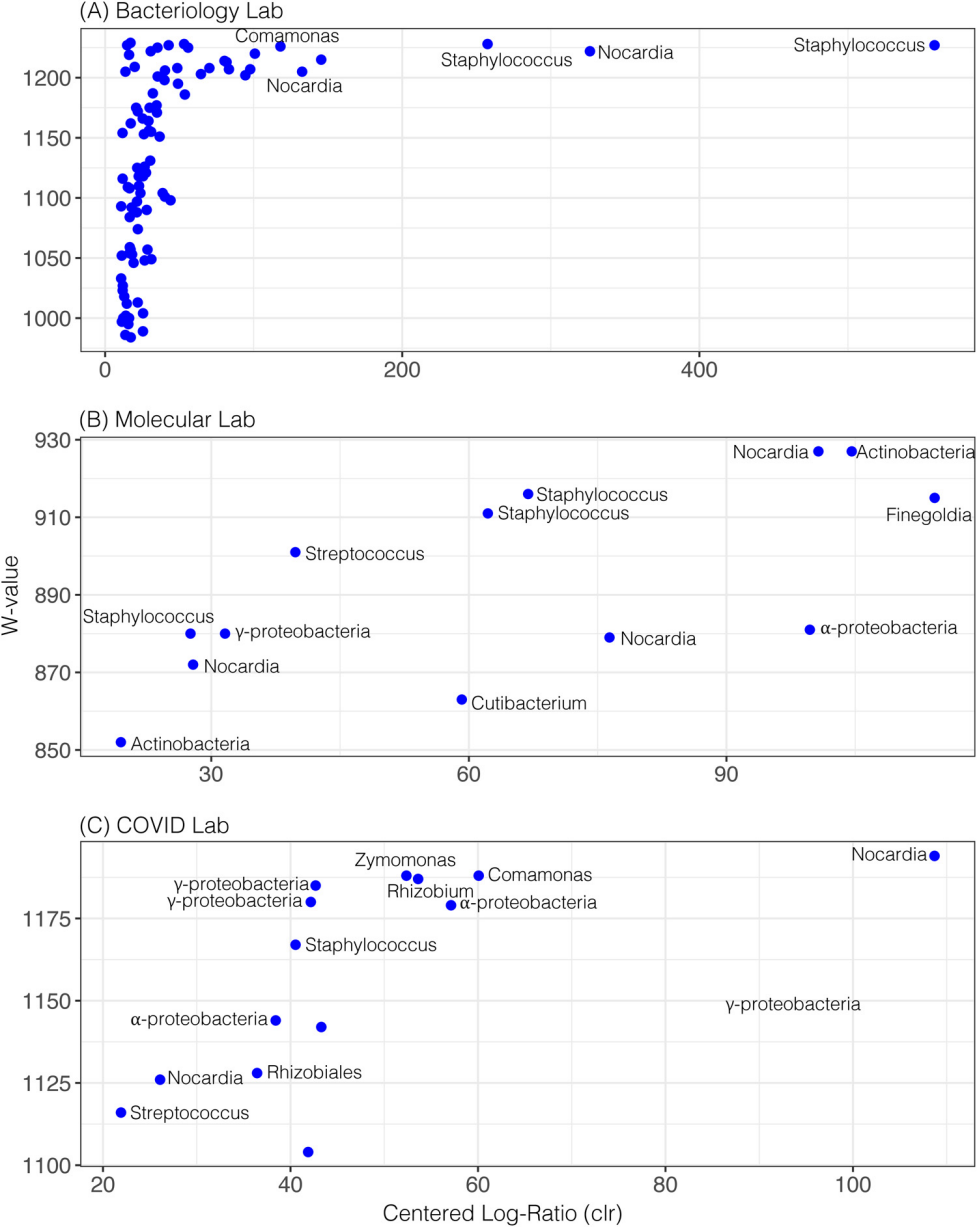
ANCOM differential abundance volcano plot. The *x* axis represents the centered log ratio (clr) transformed mean difference in abundance of a given species between sampling surfaces and the *y* axis (W-value) represents the number of times the null hypothesis (the average abundance of a taxa in a group equals to that in the other group) was rejected. Bacterial species rejecting the null hypothesis are shown in the plot above. For convenience, only 6 out of 90 species rejecting the null hypothesis are labeled in the plot A (Bacteriology lab section). Similarly, species rejecting the null hypothesis are labeled in the plot B (Molecular lab section) and plot C (COVID lab section). Only species with > 95% confidence in ANCOM analysis are shown.

We performed differential abundance analysis of the microbes found on various laboratory surfaces using the ANCOM test (60) to identify bacterial taxa that significantly differed between laboratory sections. Results are presented in the form of a volcano plot which summarizes how different a species is on one surface compared to the other. We found 90 species that were differentially abundant in the bacteriology lab section with a confidence limit of >95% (Fig. 5A), with 2 of these being different species of Staphylococcus. The presence of Staphylococcus could reflect the skin bacteria of people working in these spaces but could also represent the fact that Staphylococcus is the most common bacterium identified in cultures in this part of the laboratory. We also found that *Nocardia* and *Comamonas* were among differentially abundant taxa in the bacteriology lab section, which are common soil bacteria (61, 62), but also known to cause infections in immunocompromised individuals (63, 64). The number of differentially abundant taxa was much lower in the molecular and COVID overflow lab sections than was for the bacteriology section (Table S1). In the molecular lab section (Fig. 5B), we identified different species of Staphylococcus, Streptococcus, and *Cutibacterium*, which are common human-associated bacteria. We also found environmental bacteria such as *Nocardia* and Actinobacteria. In the COVID overflow laboratory section (Fig. 5C), which was not in operation until 10/13/2020, we did identify some Staphylococcus and Streptococcus, but more commonly identified bacteria that probably were associated with the environment, including *Nocardia*, *Rhizobium*, Rhizobiales, *Comamonas*, *Zymomonas*, and other alpha- and gamma-proteobacteria.

**FIG 6:**
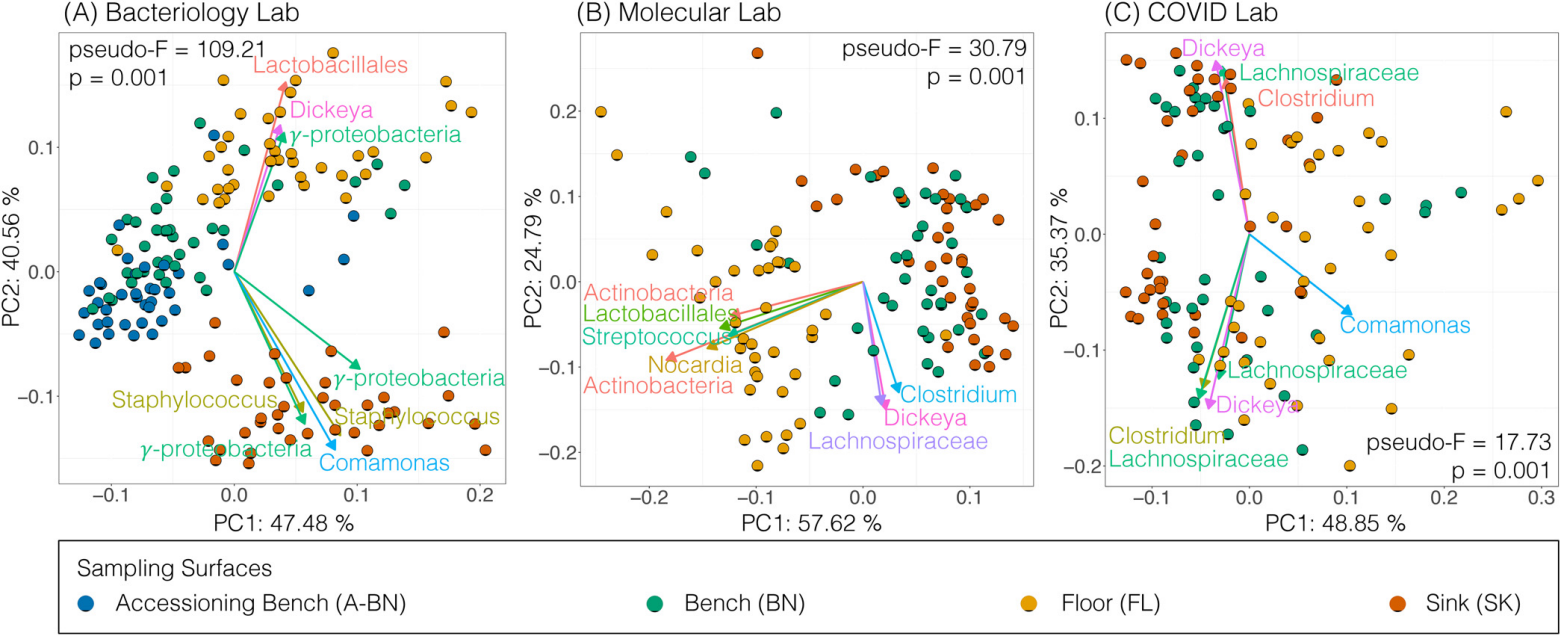
Longitudinal Beta diversity analysis. Aitchison compositional biplots for Bacteriology (A), Molecular (B), and COVID (C) lab sections. Colored dots represent individual samples from different surfaces (Accessioning Bench, Bench, Floor, and Sink). Arrows denote 8 major taxa driving the diversity within the clusters. Significance determined by PERMANOVA for individual sampling surfaces, and denoted in the corner of each panel *, *P* < 0.05.

Finally, we evaluated each laboratory section to identify bacteria that may be driving the differences between the surfaces. We performed Aitchison PCA (65), which is commonly used to identify taxa that are primarily responsible for sample clustering (66, 67). As we found using PCoA (Fig. 3), there was significant clustering of samples from different surfaces within individual laboratory sections (PERMANOVA; *P* < 0.05) (Fig. 6). We measured pseudo-F value (a measure of degree of group separation) for each laboratory section, and found that the bacteriology section (pseudo-F = 109.21) had higher degree of separation between surfaces than molecular (30.79) or COVID (17.73), which suggests that the greatest beta diversity observed was in the bacteriology section (Fig. 6). We also overlaid each PCoA with DEICODE biplots (66), which identified significant taxa responsible for driving clustering within each group. We found that many of the taxa driving diversity within the laboratory sections were associated with human skin or originated from the environment. For example, we found Staphylococcus along with environmental microbes *Dickeya*, *Comamonas*, and gamma-proteobacteria as drivers of diversity in the bacteriology section (Fig. 6A). Similarly, Streptococcus, *Comamonas*, and *Dickeya* were also identified as drivers of diversity in the molecular and COVID overflow sections, accompanied by *Clostridium*, *Nocardia*, Lachnospiraceae, and Actinobacteria (Fig. 6B and C).

## DISCUSSION

Since the inception of the pandemic in early 2020, laboratories, such as the one at UC San Diego Health, have been testing large numbers of samples for the virus that causes the disease COVID-19. Particularly early in the pandemic, there was significant concern that the virus may be harbored on laboratory surfaces and, as such, present an infection risk to the staff performing such tests. The early testing in this clinical laboratory took place before there was any mask mandate in the facility, and when the World Health Organization announced that the virus could be transmitted via an airborne fashion, there was a significant concern that such an airborne nature could result in a number of workplace exposures. While we had no reports of employees testing positive for SARS-CoV-2 during the sample collection for this study, there was still significant concern for workplace exposures. Some of the highest risk practices occurred when the facility was testing up to 4,500 specimens per day (68), which meant that not all samples could be handled in biosafety cabinets. The simple opening and closing of the caps in each tube promoted the risk of aerosolizing the live virus. It has been recently shown through RT-qPCR that SARS-CoV-2 RNA was present on surfaces of clinical microbiology laboratories indicating a possible role of environmental contamination (69). There was therefore a significant interest for us to perform such a study to identify where/if SARS-CoV-2 existed outside the collection tubes in the laboratory (70). The primary test used for detection of SARS-CoV-2 is a RT-qPCR test (71) to detect the presence of virus RNA. We observed a surge in SARS-CoV-2 on floor surfaces during the 5th week of our study. We believe that this could be linked to the large number of tests being performed and the relatively high positivity rates that we observed during that period. Additionally, it could have been related to changes in the frequency of floor cleaning during this time period. However, there was no documentation of any changes in laboratory cleaning practices during this time period. Because of the relatively chaotic atmosphere in the clinical laboratory during this time, which represented the beginnings of a surge in SARS-CoV-2 positivity rates where there were high volumes of testing and high positivity rates, we cannot be confident that there were not subtle changes in laboratory cleaning practices that were not documented. Our findings were largely reassuring that we could identify SARS-CoV-2 almost exclusively from the floors of the lab, but not from the sinks or the benches. We did, however, identify a single SARS-CoV-2 positive specimen from A-BN. All the specimens arrive in the laboratory through A-BN, and, occasionally, these specimens leak when the caps are not sufficiently secured. It is possible that the positive A-BN specimen is the result of leakage, rather than A-BN serving as a persistent risk for SARS-CoV-2 transmission since benches were regularly cleaned with a 0.5% solution (vol/vol) of sodium hypochlorite (household bleach), followed by a wipe with 70% ethanol. This helped ensure that bench surfaces did not carry viable pathogenic microbes, and, hence, reduced the chances of laboratory-acquired infections among laboratory workers.

Bacterial alpha diversity on the floors of the clinical lab section was richer and more diverse (Fig. 2) than those detected on other surfaces. A prior study had also shown that indoor floor materials serve as microbial reservoirs, especially soilborne bacteria (72). Our finding of greater diversity on the floor surfaces is also supported by previous reports showing a significant correlation between the microbiome of shoe soles and floor surfaces (73, 74). Floors come in direct contact with shoes, which are typically contaminated with microbes from environmental sources, such as soil and water. We found that there was no significant difference in alpha diversity among floor samples from all the lab sections (Fig. S1). This could potentially be governed by the microbes tracked inside on the bottoms of shoes, combined with commensal microbes already living in these spaces, which may not lead to significant variation between different lab sections.

Significant proportions of potentially environmentally-derived bacteria were present on the floors, such as Actinobacteria and Nocardia (Fig. 6). The 16S rRNA amplicon sequencing analysis could not identify specific taxa, and these microbes are generally found in the environment. While taxa associated with Actinobacteria and Nocardia have been known to cause infections (75–78), there is no evidence to suggest the organisms identified posed any threat to health care workers. However, their presence does support the need to continue strict sterile techniques and to regularly sanitize testing areas. We believe that such sanitation practices account for the substantial differences in the representation of human skin-associated organisms between the bacteriology section and other parts of the laboratory. For example, Staphylococcus and Streptococcus were among the most abundant microbes identified in the bacteriology section (Fig. 5), indicating the substantial contribution that laboratory workers likely have to the BE microbiome. However, Staphylococcus and Streptococcus are also among the most common pathogens identified in patient cultures from this section of the laboratory, so it is not clear the extent to which patient cultures contribute to the BE microbiome in this section. Particularly, where Staphylococcus is so prevalent among the patient population and the Methicillin-resistant Staphylococcus aureus (MRSA) variant of Staphylococcus is known to colonize the skin of both patients and laboratory workers. Unfortunately, a much more detailed study examining the movements of Staphylococcus throughout the lab would be necessary to decipher the relative contributions of patients and lab workers to the representation of Staphylococcus. While Staphylococcus was found at high proportions in the bacteriology section, it was less prevalent in both the molecular and COVID overflow sections (Fig. 5). One potential explanation for this is that patient cultures (which do not take place in molecular and COVID overflow sections) may have been contributing to the proportion of Staphylococcus found in the bacteriology section. We were able to identify Staphylococcus and Streptococcus in the COVID overflow laboratory, but they were of relatively low proportion, even compared to the molecular section. This difference may be due to the fact that the COVID overflow laboratory testing began after the beginning of this study and was largely unpopulated by workers for much of this time. Thus, we observed an increased proportion of environment-associated bacteria compared to human-associated bacteria in this section (Fig. 6).

Studies suggest that human skin, respiratory tract, gastrointestinal/urogenital associated bacteria, as well as those originating from water and soil habitats are the primary contributors to microbial diversity in many indoor BEs, such as restrooms (79, 80), kitchens (81), child-care facilities (53), and airplanes (82). Interestingly, the microbiota of health care facilities, including hospitals (48, 49, 83, 84), and ICUs (45, 51, 58) is remarkably similar to every other built environment. It has been reported that the most common bacteria associated with indoor surfaces belong to *Corynebacterium*, Staphylococcus, Streptococcus, *Lactobacillus*, Mycobacterium, *Bacillus*, Pseudomonas, Acinetobacter, *Sphingomonas*, *Methylobacterium*, and other members of the Enterobacteriaceae family (32, 85, 86). Like other BEs and health care facilities, we found that the most common bacteria colonizing surfaces in a clinical diagnostic laboratory primarily belong to the genera *Dickeya*, Staphylococcus, Streptococcus, *Lactobacillus*, *Nocardia*, *Comamonas*, *Clostridium*, and to members of Actinobacteria, Lachnospiraceae, and gamma-proteobacteria phyla and families. Clinical laboratories are a specialized BE that house trained medical staff, and are exposed to a plethora of human commensal and pathogenic microbes. Surfaces in the clinical laboratory, particularly the floors, come in direct contact with shoes that bring a rich source of environmental microbes into the facility. This study provides a glimpse into the complex microbiota of an important and often neglected health care-associated facility.

In this study, we characterized the complex bacterial communities inhabiting the inanimate surfaces in a diagnostic clinical laboratory during the period of SARS-CoV-2 pandemic. While the 16S rRNA gene sequencing method is the most widely used technique to explore the microbial diversity that could otherwise go unrecognized by culture alone, there are some inherent limitations. For example, variation in 16S copy number, primer binding, and amplification efficiencies can limit the accuracy in bacterial abundance and diversity estimations (87–91). Similarly, this technique without modification does not inform about the viability and infectivity of the microbes being assessed. Another important limitation is that the technique based on a small segment of 16S rRNA (V3-V4 hypervariable region) often cannot resolve taxonomic classification for some medically important bacteria. A study utilizing metagenomic sequencing and classification of the bacteria on the laboratory surfaces could potentially resolve taxonomic classifications that were limited in this study.

As far as we can tell, the study described here is the first comprehensive survey of the microbiome of a clinical microbiology laboratory. BEs, such as this one, have likely not been previously described because the connection between hospital infections and laboratory infections are not always obvious. For example, there have been well documented outbreaks of pathogen infections, such as Salmonella and *Shigella* in clinical laboratories before, and pinpointing the source of these infections back to an individual patient is usually obvious. However, when dealing with other more pervasive pathogens such as MRSA, where the source could be a myriad of patients, and the laboratory workers who acquire MRSA infections could have been infected through another means, can be quite difficult. While our analysis here will not pinpoint the source of infection in any given case, it does help to elucidate microbes that colonize these surfaces and have the potential to cause LAIs. What is most favorable about this study is that it took place during a surge in the SARS-CoV-2 pandemic when the laboratory was testing thousands of specimens per day. We had no reports of SARS-CoV-2 LAIs during this period, we do note that along with potential pathogens colonizing laboratory surfaces, there was also SARS-CoV-2 on some surfaces in the lab. This study illuminates that we did not find SARS-CoV-2 on the benches (with one exception) or in the sinks of the lab, but instead found it largely on the floors. We believe that the virus arrived on the floors largely through droplets that settled to the ground and were captured by our swabs. Because of the techniques used to identify the presence of this virus, we cannot determine whether the virus from the floors was still infectious. The relative lack of SARS-CoV-2 on the working benches suggests that basic laboratory sanitary practices can help to prevent exposures.

## MATERIALS AND METHODS

### Study site and sampling scheme in diagnostic laboratory sections.

Samples were collected from the University of California San Diego Center for Advanced Lab Medicine (UCSD CALM) in La Jolla, California. We sampled the clinical microbiology laboratory with 2 separate goals in mind. The first was to understand the succession of bacteria throughout the facility over time, and the second was to understand where the SARS-CoV-2 virus may be present on surfaces in a laboratory performing routine SARS-CoV-2 PCR testing. We collected swab samples from a defined area (~ 2 to 4 square feet) of A-BN, BN, SK, and FL surfaces (Fig. 1A and B) from each laboratory section: bacteriology, molecular microbiology, and COVID overflow within the microbiology laboratory between 07/20/2020 and 10/30/2020, coinciding with a time during which there was substantial SARS-CoV-2 transmission in the USA. The swabbing was conducted longitudinally from the same defined regions of each surface throughout the study period. Briefly, pre-moistened sterile swab (BD Swube-Dual Swab Sterile cat# 281130) were rubbed for 30 s over the surfaces. An additional site that acts as the entry point for the bacteriology lab, here called the accessioning bench, where samples are received from hospitals and clinics, was also sampled. The samples were collected from each lab section during the day shift working hours. The swabbed sink areas include the front, sides, and bottom of the sink basin, which are likely to have contact with runoff water from employees’ hands during hand washing. The swabbed floor areas include spaces near the working benches. Collected samples were stored at − 80°C for around 6 months before processing.

### Nucleic acid extraction and high-throughput DNA sequencing.

The collected swabs were homogenized in sterile phosphate buffered saline (PBS) buffer for 10 min, followed by brief vortexing to dislodge bacteria and any particulate matter from the swabs. A total of 500 μL resuspended PBS buffer from the tube was subjected to total nucleic acid (DNA/RNA) extraction and concentration using PureLink Viral RNA/DNA minikit (Invitrogen, cat# 12280050) and Zymo gDNA Clean and Concentrator-10 kit (Zymo research cat# 11-316), respectively. Additionally, unused sterile swabs were used for negative control during the extraction to ensure no DNA contamination occurred during the process. The nucleic acid was subjected for PCR amplification of V3-V4 hypervariable region of the 16S rRNA gene using Kapa Hifi Hotstart Readymix (Kapa Biosystems; KK2602) with forward primer 5′-TCG TCG GCA GCG TCA GAT GTG TAT AAG AGA CAG CCT ACG GGN GGC WGC AG-3′ and reverse primer 5′-GTC TCG TGG GCT CGG AGA TGT GTA TAA GAG ACA GGA CTA CHV GGG TAT CTA ATC C-3′ (92) using the following cycling parameters: 95°C for 3 min, followed by 35 cycles of 95°C for 30 s, 55°C for 30 s, 72°C for 30 s, and a final elongation step of 72°C for 5 min. Ampure XP beads (Beckman-Coulter Life Sciences product# A63881) were used to clean PCR amplicons. The cleaned PCR amplicons were indexed using Nextera XT index kit v2 (Illumina), followed by cleaning by Ampure XP beads. Indexed and cleaned PCR products were then analyzed using High Sensitivity DNA Chips (Agilent Technologies part# 5067-4626) on an Agilent 2100 Bioanalyzer (Agilent Technologies) and quantified using Qubit dsDNA HS assay kit (Invitrogen cat# Q32851) on a Qubit 2.0 Fluorometer (Thermo Fisher Scientific). Finally, samples were normalized and pooled into equal molar concentration and sequenced using MiSeq reagent kit v3 (600-cycle) on the Illumina MiSeq platform (Illumina).

### Realtime qPCR for COVID screening.

Samples were tested for the presence of SARS-CoV-2 viral RNA using 7500 Fast Dx Real-Time PCR instrument (Thermo Fisher Scientific) and the TaqPath COVID-19 Combo Kit (Thermo Fisher Scientific). The MagMax Viral/Pathogen Nucleic Acid isolation kit was used for nucleic acid extraction in conjunction with the KingFisher Flex Instrument (Thermo Fisher Scientific). A 25 μL reaction was prepared for qualitative detection of SARS-CoV-2 by RT-qPCR utilizing 5 μL of MagMax extracted RNA, 15 μL of reaction mix, which included TaqPath 1-Step Multiplex Master Mix and COVID-19 Real-Time PCR Assay Multiplex reagent (Thermo Fisher Scientific), which included primers and probe sequences targeting ORF1ab, N Protein, and S Protein, according to manufacturer’s instructions. Thermal cycling was performed at 25°C for 2 min, 53°C for 10 min for reverse transcription, followed by 95°C for 2 min, and then 40 cycles of 95°C for 3 s, and 60°C for 30 s. The cut-off threshold (*C*_t_ value) for each viral target to be considered positive was ≤ 37, and at least 2 of the 3 genes had to be detected. Some specimens tested were indeterminate (N = 26) and had to be repeated. Samples were considered indeterminate if only 1 out of 3 genes were detected. Upon repeat, only 4 specimens remained indeterminate, and were excluded from the study.

### 16S rRNA gene sequencing analysis.

We sequenced a total of 4,449,418 reads from 329 swab samples over the 15-week course of this study. There were approximately 17,813 reads per specimen with a median of 10,799 per sample. For further analysis, we chose a sampling depth parameter of 6,469 sequence reads, which retained 2,128,301 (50.00%) reads in 329 (86.58%) samples which were grouped by laboratory section (130 for bacteriology, 100 for molecular, and 99 for COVID), or by sampling surfaces (33 for accessioning bench, 99 for benches, 94 for floors, and 103 for sinks).

Sequenced reads were processed with Quantitative Insights Into Microbial Ecology 2 (QIIME2; version 2021.4) (93). The Deblur plugin in QIIME2 was used for quality filtering and denoising the data (94). Taxonomy classification was generated using the QIIME feature-classifier, with Naive Bayes classifiers trained on the SILVA database (version 138) (95). Alpha diversity was analyzed using Observed Amplicon Sequence Variants (ASVs), Faith’s Phylogenetic Diversity (96), and Shannon Index (97) as measures of alpha diversity metrics and beta diversity was measured using Bray Curtis diversity metric by QIIME2 core-metrics phylogenetic pipeline (sampling depth parameter 6,469). Additionally, due to the compositional nature of the data, we implemented a quantitative beta diversity metric, robust Aitchison PCA, using the DEICODE plugin with taxonomic biplot overlays (66). The results were imported and visualized using the qiime2R (available at https://github.com/jbisanz/qiime2R) and ggplot2 packages in R-Studio (version 1.4.1717) (98). Comparison of the relative abundances at the family level was visualized using ggplot2 with the exported QIIME2 taxonomy tables. The QIIME2 core-feature function, with the maximum fraction set to 90%, was used to define the “core microbiome” for each of the sample sources (Bench, Floor, and Sink), which identified the features (e.g., amplicon sequence variant) present in at least 90% of samples from each source. Finally, the Analysis of Compositions of Microbiomes (ANCOM) test in the q2-composition plugin was used to assess the differential abundance of the bacterial taxa at genus level (60).

### Statistical tests.

Statistical significance for alpha diversity among sampling sites were determined using pairwise Kruskal-Wallis test. Pearson correlation coefficients were calculated in R-Studio (2022.02.1) to identify associations between the sampling days and the alpha diversity metric (Shannon Index). Beta diversity significance was determined using ANOSIM tests with 999 permutations in R-studio, and the significance of the Aitchison PCA was quantified by the PERMANOVA test with 999 permutations using DEICODE plugin in QIIME2.

### Data availability.

All sequences included in this study have been deposited in the NCBI Sequence Read Archive under the BioProject accession PRJNA812962.
