# Supplementary material for: Characterization of SARS-CoV-2 Distribution and Microbial Succession in a Clinical Microbiology Testing Facility during the SARS-CoV-2 Pandemic
Source: Microbiol Spectr. 2023 Mar 14;11(2):e04509-22. doi: 10.1128/spectrum.04509-22 (PMC10100919; doi:10.1128/spectrum.04509-22)
Supplement: Supplemental file 1 — Supplemental material. Download spectrum.04509-22-s0001.pdf, PDF file, 1.2 MB [file spectrum.04509-22-s0001.pdf]

Supplementary Material

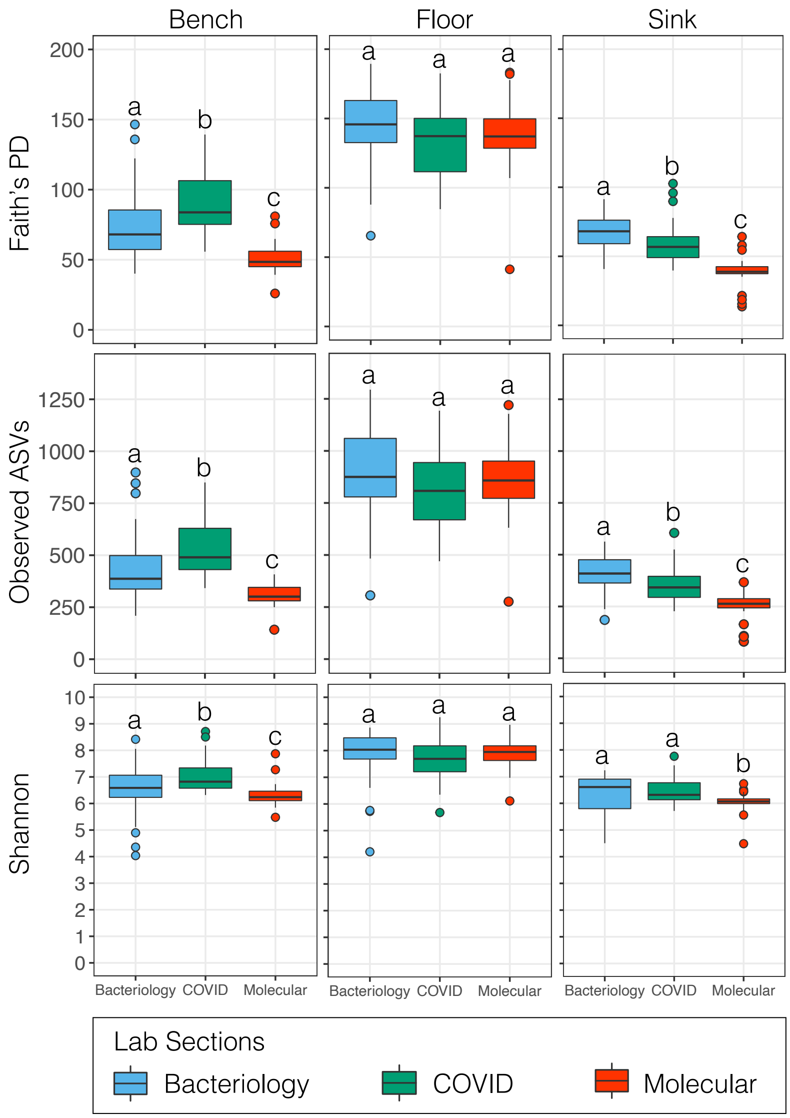

Fig S1. Alpha diversity boxplot showing Faith's PD (top panel), Observed ASV (middle panel) and Shannon's index (bottom panel) for sampling surfaces with samples grouped by lab sections. Alpha diversity metrics (Faith's PD, Observed ASV and Shannon) are shown on the y-axis in different panels, while lab section groups are shown on the x-axis. Letters ("a", "b", "c", "d") shared in common between lab sections for individual sampling surfaces indicate no significant difference ($p_{adj} > 0.05$) as determined by the Kruskal-Wallis test. For example, lab sections from Floor do not differ from each other (Faith's PD, Observed ASV and Shannon) while from Bench differ significantly. The boxplots show the Interquartile Range (IQR) between the first and third quartiles, the center line is the median, Whiskers represent the smallest (y-min) and largest (y-max) observations within 1.5 times the IQR from the first and third quartiles respectively. Outliers indicated by respective colored circles.

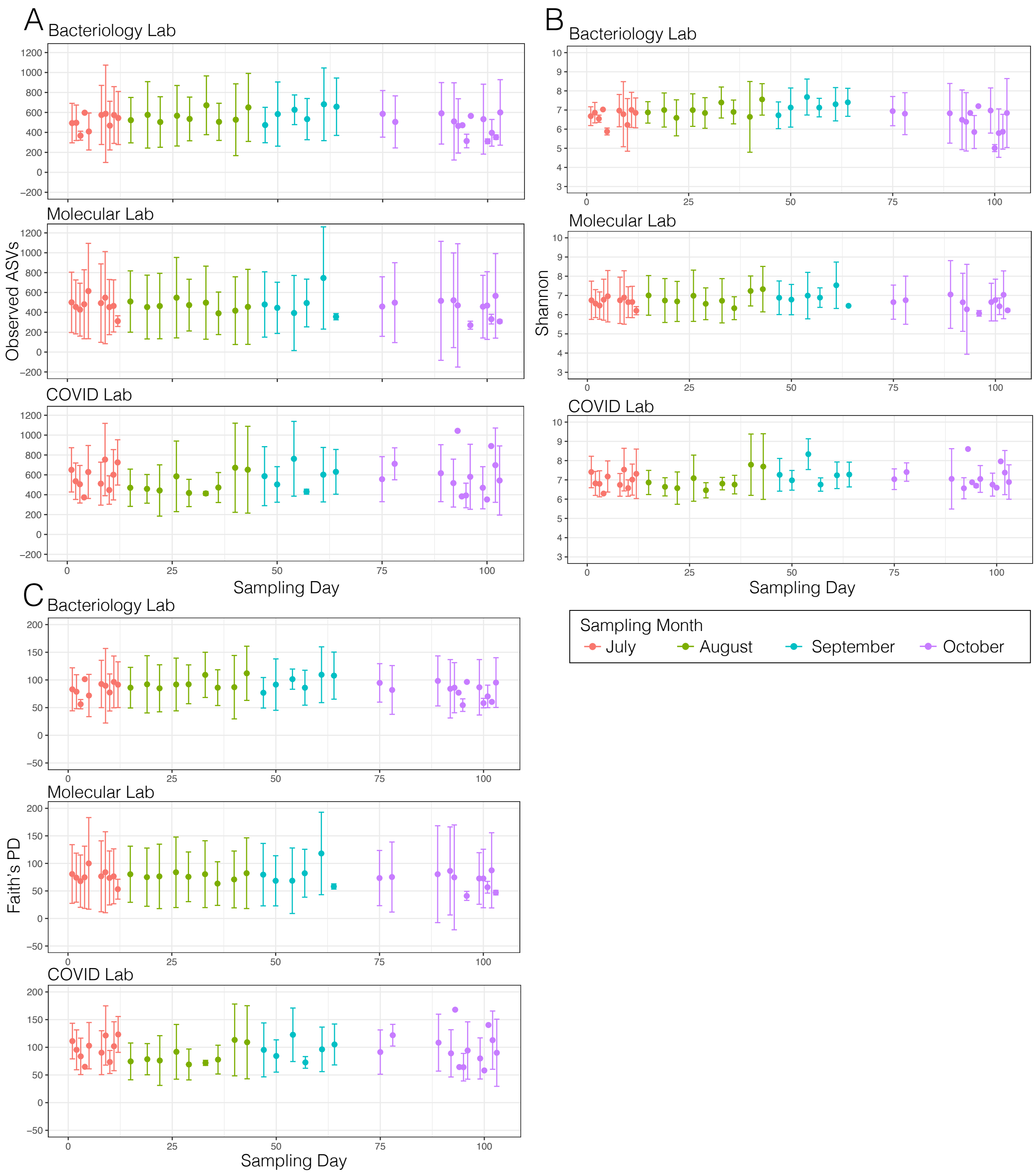

Fig S2. Dotplot of the (A) Observed ASVs, (B) Shannon and (C) Faith's PD for samples collected from Bacteriology, Molecular and COVID lab sections. For each sampling day, the mean number (± standard deviation) of the samples collected on individual sampling days are shown on the y-axis and the sampling days are on the x-axis. Different colors represent samples grouped into four different months.

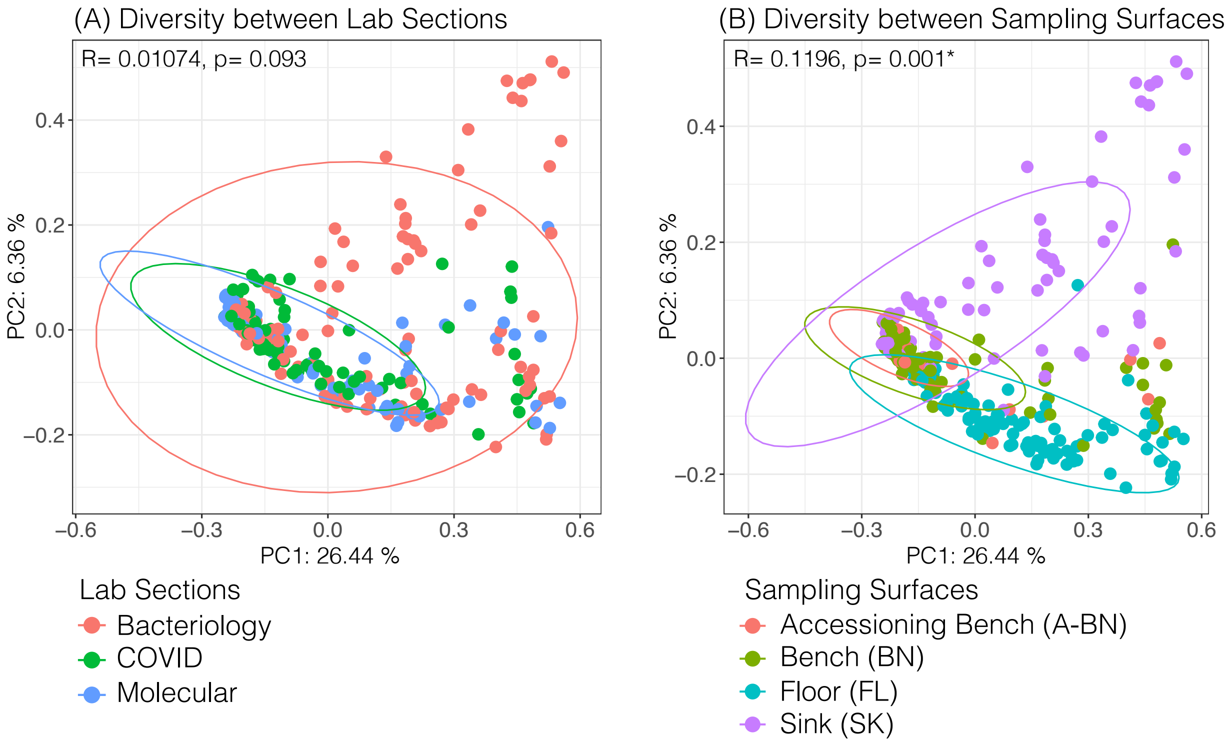

Fig S3. Principal coordinates analysis of beta diversity based on Bray Curtis dissimilarities by lab sections (A) and sampling surfaces (B). Ellipses are drawn at 95% confidence intervals for each group. Significance determined by ANOSIM with 999 permutations for individual lab and denoted in the corner of each panel $*p < 0.05$.

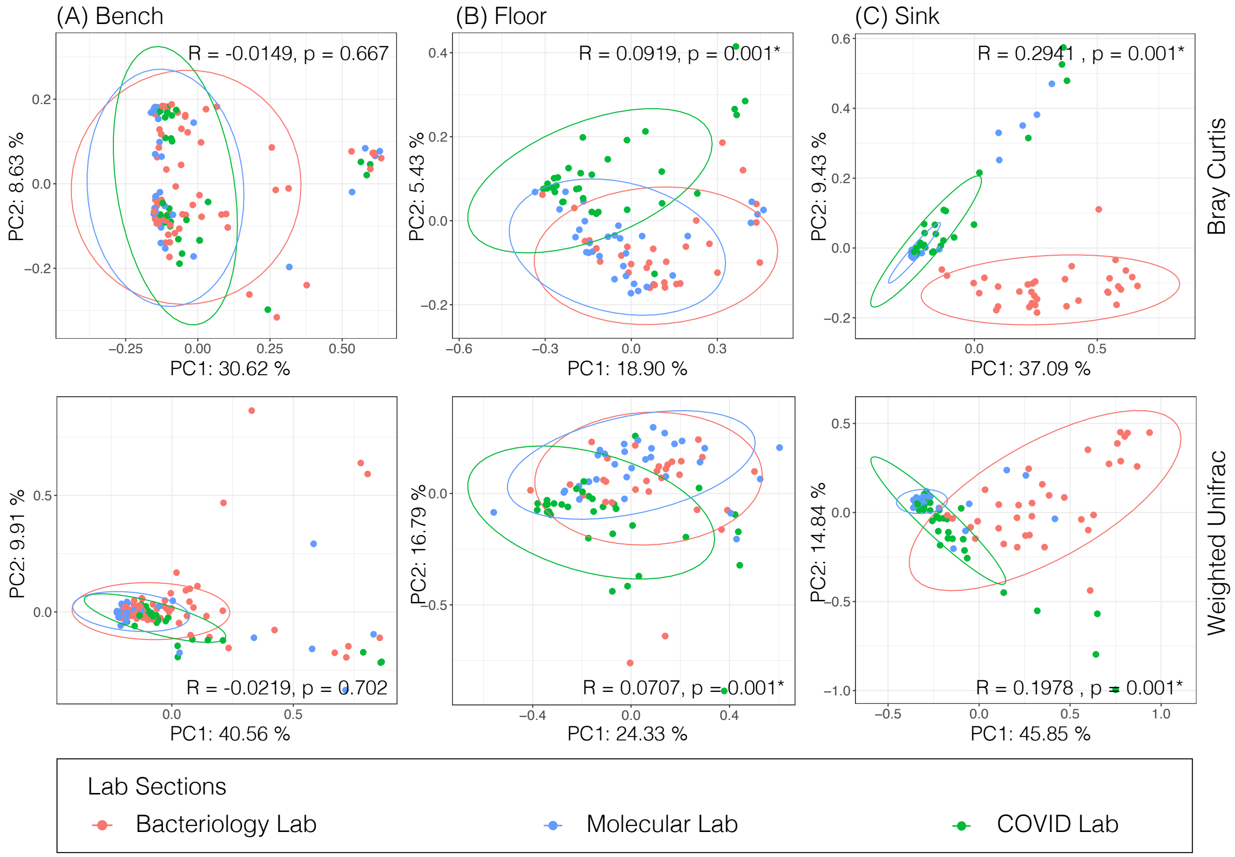

Fig S4. Principal coordinates analysis (PCoA) plot based on Bray Curtis and Weighted Unifrac dissimilarities depicting the clusters of bacterial communities for Bench (A), Floor (B), and Sink (C) grouped by lab sections. Ellipses are drawn at 95% confidence intervals and significance was determined by ANOSIM with 999 permutations for individual lab sections and denoted in the corner of each panel $*p < 0.05$.

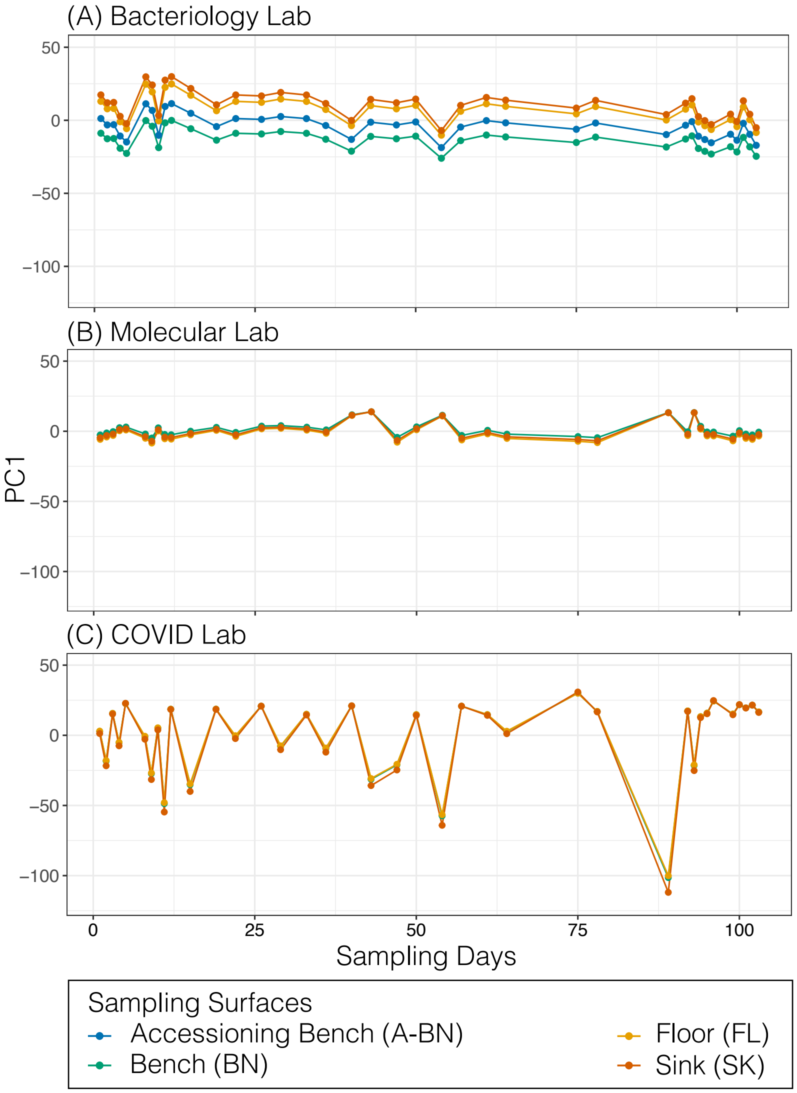

Fig S5. Beta-diversity analyses showing diversity between samples collected from different sampling surfaces across time (Sampling Days). The first principal component (PC1) from compositional tensor factorization (CTF) is plotted on the y axis with the corresponding sampling day on the x-axis.

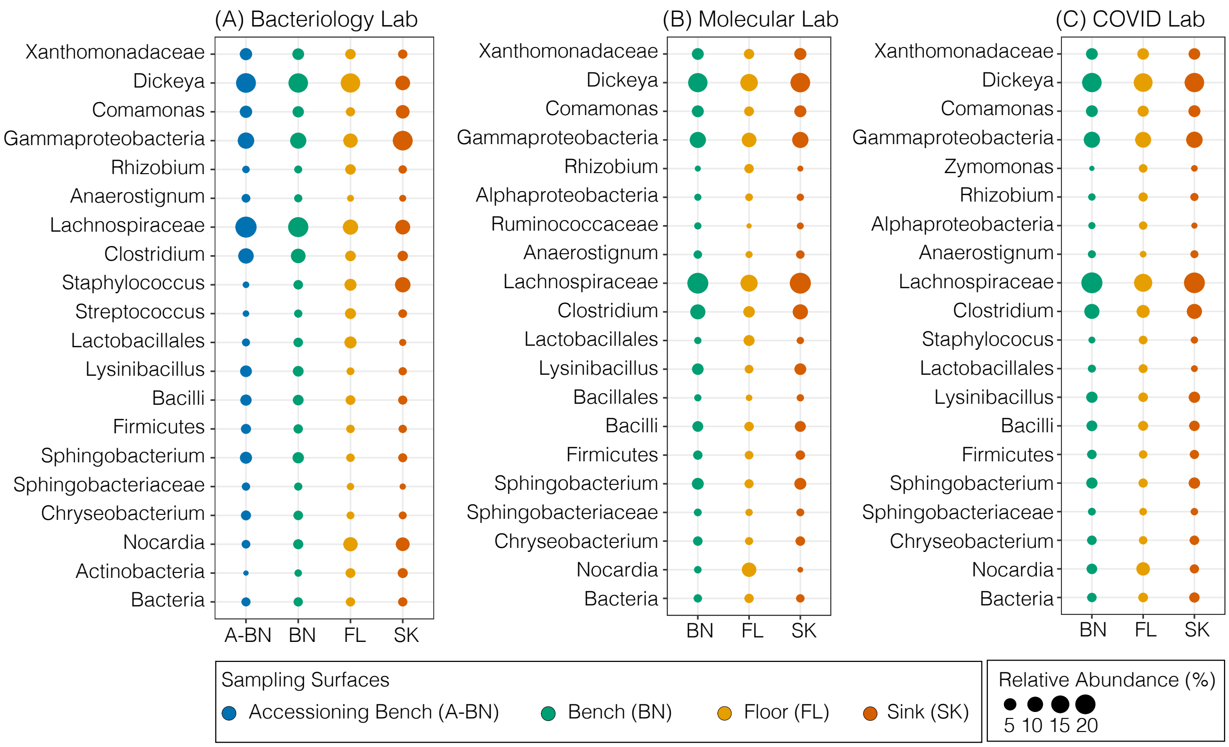

Fig S6. Dotplot of the percentage relative abundances of the 20 most abundant bacterial taxa on different sampling surfaces from the bacterial, molecular and COVID lab sections. Median values were calculated for relative abundance on collapsed feature table at the genus level (i.e., level 6). For each sample source, (Accessioning Bench, Bench, Floor and Sink) the taxa determined to be significantly different between surfaces are shown on the y-axis and the sampling surfaces are on the x-axis. The size of the dot reflects the median relative abundance of each taxon and the color denotes the sampling surfaces.

Table S1. Differentially abundant bacterial taxa within individual lab sections (ANCOM test)

SN	Observed Taxonomic Units	Confidence	clr	W-value	Reject null hypothesis
	Bacteriology Lab				
1	d__Bacteria; p__Firmicutes; c__Bacilli; o__Staphylococcales; f__Staphylococcaceae; g__Staphylococcus; s__Staphylococcus_carnosus	0.999883783	558.2663948	1227	TRUE
2	d__Bacteria; p__Actinobacteriota; c__Actinobacteria; o__Corynebacteriales; f__Nocardiaceae; g__Nocardia; s__Streptomyces_sp.	0.984048797	326.3338946	1222	TRUE
3	d__Bacteria; p__Firmicutes; c__Bacilli; o__Staphylococcales; f__Staphylococcaceae; g__Staphylococcus; s__Staphylococcus_carnosus	0.999757651	257.4273242	1228	TRUE
4	d__Bacteria	0.999984214	145.4500964	1215	TRUE
5	d__Bacteria; p__Actinobacteriota; c__Actinobacteria; o__Corynebacteriales; f__Nocardiaceae; g__Nocardia; s__Streptomyces_sp.	0.999998955	132.6016272	1205	TRUE
6	d__Bacteria; p__Proteobacteria; c__Gammaproteobacteria; o__Burkholderiales; f__Comamonadaceae; g__Comamonas; s__uncultured_compost	0.993383633	118.0030956	1226	TRUE
7	d__Bacteria; p__Proteobacteria; c__Gammaproteobacteria	0.976493893	100.806751	1220	TRUE
8	d__Bacteria; p__Actinobacteriota; c__Actinobacteria; o__Corynebacteriales; f__Nocardiaceae; g__Nocardia; s__Streptomyces_sp.	0.99999993	97.65385012	1207	TRUE
9	d__Bacteria; p__Proteobacteria; c__Alphaproteobacteria; o__Rhizobiales; f__Rhizobiaceae; g__Allorhizobium-Neorhizobium-Pararhizobium-Rhizobium; s__[Pseudomonas]_geniculata	0.998874025	94.39048819	1202	TRUE
10	d__Bacteria; p__Firmicutes; c__Bacilli; o__Staphylococcales; f__Staphylococcaceae; g__Staphylococcus; s__Staphylococcus_carnosus	0.999708121	83.35275262	1207	TRUE
11	d__Bacteria; p__Proteobacteria; c__Gammaproteobacteria; o__Burkholderiales; f__Comamonadaceae; g__Comamonas; s__uncultured_compost	0.991786763	81.75043552	1213	TRUE
12	d__Bacteria; p__Actinobacteriota; c__Actinobacteria; o__Corynebacteriales; f__Nocardiaceae; g__Nocardia; s__Streptomyces_sp.	0.997352966	80.36034444	1214	TRUE
13	d__Bacteria; p__Firmicutes; c__Bacilli; o__Staphylococcales; f__Staphylococcaceae; g__Staphylococcus; s__Staphylococcus_carnosus	0.999736239	70.19390129	1208	TRUE
14	d__Bacteria; p__Proteobacteria; c__Gammaproteobacteria	0.98940857	64.49920445	1203	TRUE

15	d__Bacteria; p__Firmicutes; c__Bacilli; o__Staphylococcales; f__Staphylococcaceae; g__Staphylococcus; s__Staphylococcus_carnosus	0.999991447	55.866 90322	1225	TRUE
16	d__Bacteria; p__Actinobacteriota; c__Actinobacteria; o__Corynebacteriales; f__Nocardiaceae; g__Nocardia; s__Streptomyces_sp.	0.972053371	53.679 17906	1186	TRUE
17	d__Bacteria; p__Firmicutes; c__Bacilli; o__Staphylococcales; f__Staphylococcaceae; g__Staphylococcus; s__Staphylococcus_carnosus	0.999981875	53.184 56004	1228	TRUE
18	d__Bacteria; p__Proteobacteria; c__Alphaproteobacteria	0.999783711	49.065 2853	1195	TRUE
19	d__Bacteria; p__Proteobacteria; c__Alphaproteobacteria; o__Sphingomonadales; f__Sphingomonadaceae	0.955703315	48.581 39212	1208	TRUE
20	d__Bacteria	0.999973745	44.041 2835	1098	TRUE
21	d__Bacteria; p__Actinobacteriota; c__Actinobacteria	0.999964041	42.766 49405	1227	TRUE
22	d__Bacteria; p__Proteobacteria; c__Gammaproteobacteria	0.994014897	40.280 48797	1206	TRUE
23	d__Bacteria; p__Actinobacteriota; c__Actinobacteria; o__Corynebacteriales; f__Nocardiaceae; g__Nocardia; s__Streptomyces_sp.	0.999998375	40.228 3821	1101	TRUE
24	d__Bacteria; p__Firmicutes; c__Bacilli; o__Staphylococcales; f__Staphylococcaceae; g__Staphylococcus; s__Staphylococcus_carnosus	0.999978572	39.983 89737	1198	TRUE
25	d__Bacteria; p__Actinobacteriota; c__Actinobacteria; o__Corynebacteriales; f__Nocardiaceae; g__Nocardia; s__Streptomyces_sp.	0.999978453	38.736 23285	1104	TRUE
26	d__Bacteria; p__Actinobacteriota; c__Actinobacteria; o__Corynebacteriales; f__Nocardiaceae; g__Nocardia; s__Streptomyces_sp.	0.999754083	36.713 2329	1151	TRUE
27	d__Bacteria; p__Proteobacteria; c__Gammaproteobacteria	0.985012075	35.366 85241	1201	TRUE
28	d__Bacteria; p__Proteobacteria; c__Gammaproteobacteria	0.996096408	35.248 36765	1225	TRUE
29	d__Bacteria; p__Proteobacteria; c__Alphaproteobacteria; o__Sphingomonadales; f__Sphingomonadaceae	0.953687411	34.880 2579	1171	TRUE
30	d__Bacteria; p__Actinobacteriota; c__Actinobacteria; o__Corynebacteriales; f__Nocardiaceae; g__Nocardia; s__Streptomyces_sp.	0.999095731	34.647 26986	1177	TRUE
31	d__Bacteria; p__Proteobacteria; c__Gammaproteobacteria; o__Burkholderiales; f__Comamonadaceae; g__Comamonas; s__uncultured_compost	0.993176388	32.132 86691	1187	TRUE
32	d__Bacteria	0.99997475	31.137 40878	1049	TRUE

33	d__Bacteria; p__Proteobacteria; c__Alphaproteobacteria; o__Sphingomonadales; f__Sphingomonadaceae; g__Zymomonas; s__Zymomonas_mobilis	0.950187593	30.95887046	1155	TRUE
34	d__Bacteria; p__Proteobacteria; c__Gammaproteobacteria; o__Enterobacterales; f__Pectobacteriaceae; g__Dickeya; s__Dickeya_phage	0.999938454	30.6953141	1222	TRUE
35	d__Bacteria; p__Proteobacteria; c__Alphaproteobacteria; o__Rhizobiales; f__Rhizobiaceae; g__Allorhizobium-Neorhizobium-Pararhizobium-Rhizobium; s__[Pseudomonas]_geniculata	0.999599629	30.37482495	1131	TRUE
36	d__Bacteria; p__Proteobacteria; c__Gammaproteobacteria	0.975962589	29.94621178	1175	TRUE
37	d__Bacteria; p__Actinobacteriota; c__Actinobacteria; o__Corynebacteriales; f__Nocardiaceae; g__Nocardia; s__Streptomyces_sp.	0.999946432	29.47573969	1156	TRUE
38	d__Bacteria; p__Firmicutes; c__Bacilli	0.988566621	29.19861259	1164	TRUE
39	d__Bacteria	0.999974292	28.52384409	1057	TRUE
40	d__Bacteria; p__Proteobacteria; c__Alphaproteobacteria; o__Rhizobiales; f__Rhizobiaceae; g__Allorhizobium-Neorhizobium-Pararhizobium-Rhizobium; s__[Pseudomonas]_geniculata	0.99308386	27.97510954	1090	TRUE
41	d__Bacteria; p__Actinobacteriota; c__Actinobacteria; o__Corynebacteriales; f__Nocardiaceae; g__Nocardia; s__Streptomyces_sp.	0.997333354	27.53333086	1121	TRUE
42	d__Bacteria; p__Actinobacteriota; c__Actinobacteria; o__Corynebacteriales; f__Nocardiaceae; g__Nocardia; s__Streptomyces_sp.	0.999946849	26.60861023	1048	TRUE
43	d__Bacteria	0.999979095	26.60585589	1126	TRUE
44	d__Bacteria; p__Proteobacteria; c__Gammaproteobacteria; o__Burkholderiales; f__Comamonadaceae; g__Comamonas; s__uncultured_compost	0.991747628	26.11597494	1153	TRUE
45	d__Bacteria; p__Actinobacteriota; c__Actinobacteria; o__Corynebacteriales; f__Nocardiaceae; g__Nocardia; s__Streptomyces_sp.	0.999972971	25.55309933	1118	TRUE
46	d__Bacteria; p__Proteobacteria; c__Alphaproteobacteria	0.997621302	25.54291704	1004	TRUE
47	d__Bacteria; p__Proteobacteria; c__Alphaproteobacteria; o__Rhizobiales; f__Rhizobiaceae; g__Allorhizobium-Neorhizobium-Pararhizobium-Rhizobium; s__[Pseudomonas]_geniculata	0.993998891	25.46505128	989	TRUE

48	d__Bacteria; p__Proteobacteria; c__Gammaproteobacteria	0.975958028	25.33123465	1166	TRUE
49	d__Bacteria; p__Actinobacteriota; c__Actinobacteria; o__Corynebacteriales; f__Nocardiaceae; g__Nocardia; s__Streptomyces_sp.	0.982918638	23.76053934	1104	TRUE
50	d__Bacteria; p__Actinobacteriota; c__Actinobacteria; o__Corynebacteriales; f__Nocardiaceae; g__Nocardia; s__Streptomyces_sp.	0.993605768	22.83441412	1110	TRUE
51	d__Bacteria; p__Actinobacteriota; c__Actinobacteria; o__Corynebacteriales; f__Nocardiaceae; g__Nocardia; s__Streptomyces_sp.	0.993805888	22.76035219	1110	TRUE
52	d__Bacteria; p__Firmicutes; c__Bacilli; o__Staphylococcales; f__Staphylococcaceae; g__Staphylococcus; s__Staphylococcus_carnosus	0.999872539	22.62139228	1118	TRUE
53	d__Bacteria; p__Proteobacteria; c__Alphaproteobacteria; o__Rhizobiales; f__Rhizobiaceae; g__Allorhizobium-Neorhizobium-Pararhizobium-Rhizobium; s__[Pseudomonas]_geniculata	0.99712791	22.02477493	1074	TRUE
54	d__Bacteria; p__Proteobacteria; c__Gammaproteobacteria	0.99354253	21.97854981	1172	TRUE
55	d__Bacteria; p__Proteobacteria; c__Alphaproteobacteria; o__Rhizobiales; f__Rhizobiaceae; g__Allorhizobium-Neorhizobium-Pararhizobium-Rhizobium; s__[Pseudomonas]_geniculata	0.998696314	21.95762869	1013	TRUE
56	d__Bacteria; p__Proteobacteria; c__Alphaproteobacteria; o__Rhizobiales	0.996522166	21.59593261	1125	TRUE
57	d__Bacteria; p__Actinobacteriota; c__Actinobacteria; o__Corynebacteriales; f__Nocardiaceae; g__Nocardia; s__Streptomyces_sp.	0.988526569	21.45297763	1097	TRUE
58	d__Bacteria; p__Proteobacteria; c__Gammaproteobacteria	0.968060705	21.31851647	1088	TRUE
59	d__Bacteria; p__Actinobacteriota; c__Actinobacteria; o__Corynebacteriales; f__Nocardiaceae; g__Nocardia; s__Streptomyces_sp.	0.99996539	20.8816109	1175	TRUE
60	d__Bacteria; p__Actinobacteriota; c__Actinobacteria; o__Propionibacteriales; f__Propionibacteriaceae; g__Cutibacterium	0.965318867	19.92817045	1209	TRUE
61	d__Bacteria; p__Firmicutes; c__Clostridia; o__Lachnospirales; f__Lachnospiraceae	0.99473203	19.17256988	1046	TRUE
62	d__Bacteria; p__Actinobacteriota; c__Actinobacteria; o__Corynebacteriales; f__Nocardiaceae; g__Nocardia; s__Streptomyces_sp.	0.963200207	18.15753005	1053	TRUE
63	d__Bacteria; p__Proteobacteria; c__Gammaproteobacteria	0.9898368	17.87128375	1092	TRUE

64	d__Bacteria; p__Actinobacteriota; c__Actinobacteria; o__Corynebacteriales; f__Nocardiaceae; g__Nocardia; s__Streptomyces_sp.	0.999851727	17.25012714	984	TRUE
65	d__Bacteria; p__Firmicutes; c__Bacilli; o__Bacillales; f__Planococcaceae; g__Lysinibacillus; s__Lysinibacillus_xylanilyticus	0.970033286	17.24421059	1162	TRUE
66	d__Bacteria; p__Actinobacteriota; c__Actinobacteria; o__Corynebacteriales; f__Nocardiaceae; g__Nocardia; s__Streptomyces_sp.	0.999999725	17.15051873	1057	TRUE
67	d__Bacteria; p__Firmicutes; c__Bacilli; o__Staphylococcales; f__Staphylococcaceae; g__Staphylococcus; s__Staphylococcus_carnosus	0.999979478	17.09352886	1229	TRUE
68	d__Bacteria; p__Actinobacteriota; c__Actinobacteria; o__Corynebacteriales; f__Nocardiaceae; g__Nocardia; s__Streptomyces_sp.	0.995682736	16.59957597	1059	TRUE
69	d__Bacteria; p__Proteobacteria; c__Gammaproteobacteria	0.984796294	16.53632278	1084	TRUE
70	d__Bacteria; p__Proteobacteria; c__Alphaproteobacteria	0.99984206	16.45319521	1054	TRUE
71	d__Bacteria; p__Firmicutes; c__Clostridia; o__Peptostreptococcales-Tissierellales; f__Peptostreptococcales-Tissierellales; g__Finegoldia	0.997932772	16.43928228	1108	TRUE
72	d__Bacteria; p__Chloroflexi; c__Chloroflexia; o__Thermomicrobiales; f__JG30-KF-CM45; g__JG30-KF-CM45; s__uncultured_Thermomicrobium	0.994600293	16.1544284	1000	TRUE
73	d__Bacteria; p__Firmicutes; c__Bacilli; o__Staphylococcales; f__Staphylococcaceae; g__Staphylococcus; s__Staphylococcus_carnosus	0.999968473	16.13534592	1219	TRUE
74	d__Bacteria; p__Proteobacteria; c__Gammaproteobacteria; o__Xanthomonadales; f__Xanthomonadaceae	0.966605108	15.64495753	995	TRUE
75	d__Bacteria; p__Proteobacteria; c__Gammaproteobacteria	0.994951779	15.26944801	1109	TRUE
76	d__Bacteria; p__Firmicutes; c__Bacilli; o__Staphylococcales; f__Staphylococcaceae; g__Staphylococcus; s__Staphylococcus_carnosus	0.999990264	14.81500202	1227	TRUE
77	d__Bacteria; p__Actinobacteriota; c__Actinobacteria; o__Corynebacteriales; f__Nocardiaceae; g__Nocardia; s__Streptomyces_sp.	0.999999928	14.59671328	1012	TRUE
78	d__Bacteria; p__Actinobacteriota; c__Actinobacteria; o__Corynebacteriales; f__Nocardiaceae; g__Nocardia; s__Streptomyces_sp.	0.992173688	13.9351701	1002	TRUE
79	d__Bacteria; p__Firmicutes; c__Clostridia; o__Lachnospirales; f__Lachnospiraceae	0.997393627	13.68648682	1205	TRUE
80	d__Bacteria; p__Proteobacteria; c__Gammaproteobacteria	0.998619939	13.61752838	986	TRUE

81	d__Bacteria; p__Actinobacteriota; c__Actinobacteria; o__Corynebacteriales; f__Nocardiaceae; g__Nocardia; s__Streptomyces_sp.	0.996386115	12.878 52405	1018	TRUE
82	d__Bacteria; p__Firmicutes; c__Bacilli; o__Staphylococcales; f__Staphylococcaceae; g__Staphylococcus; s__Staphylococcus_carnosus	0.999928465	12.201 98027	1000	TRUE
83	d__Bacteria; p__Firmicutes; c__Bacilli; o__Lactobacillales; f__Streptococcaceae; g__Streptococcus; s__Streptococcus_pneumoniae	0.958647813	11.897 03728	1027	TRUE
84	d__Bacteria; p__Proteobacteria; c__Alphaproteobacteria; o__Sphingomonadales; f__Sphingomonadaceae	0.967551035	11.799 88328	1023	TRUE
85	d__Bacteria; p__Proteobacteria; c__Gammaproteobacteria; o__Enterobacterales; f__Pectobacteriaceae; g__Dickeya; s__Dickeya_phage	0.999998069	11.790 62238	1116	TRUE
86	d__Bacteria; p__Firmicutes; c__Bacilli; o__Lactobacillales; f__Streptococcaceae; g__Streptococcus; s__Streptococcus_pneumoniae	0.991288542	11.723 57442	1154	TRUE
87	d__Bacteria; p__Firmicutes; c__Bacilli; o__Lactobacillales; f__Streptococcaceae; g__Streptococcus; s__Streptococcus_pneumoniae	0.98562384	11.266 19523	997	TRUE
88	d__Bacteria; p__Actinobacteriota; c__Actinobacteria	0.999947451	11.208 44103	1052	TRUE
89	d__Bacteria; p__Firmicutes; c__Bacilli; o__Staphylococcales; f__Staphylococcaceae; g__Staphylococcus; s__Staphylococcus_carnosus	0.999975649	10.823 84657	1093	TRUE
90	d__Bacteria; p__Proteobacteria; c__Gammaproteobacteria	0.991544842	10.789 26298	1033	TRUE
	Molecular Lab				
1	d__Bacteria; p__Firmicutes; c__Clostridia; o__Peptostreptococcales-Tissierellales; f__Peptostreptococcales-Tissierellales; g__Finegoldia	0.997932772	114.24 46013	915	TRUE
2	d__Bacteria; p__Actinobacteriota; c__Actinobacteria	0.999964041	104.59 02477	927	TRUE
3	d__Bacteria; p__Actinobacteriota; c__Actinobacteria; o__Corynebacteriales; f__Nocardiaceae; g__Nocardia; s__Streptomyces_sp.	0.99996539	100.70 7447	927	TRUE
4	d__Bacteria; p__Proteobacteria; c__Alphaproteobacteria	0.999783711	99.709 88372	881	TRUE
5	d__Bacteria; p__Actinobacteriota; c__Actinobacteria; o__Corynebacteriales; f__Nocardiaceae; g__Nocardia; s__Streptomyces_sp.	0.999946432	76.377 14363	879	TRUE
6	d__Bacteria; p__Firmicutes; c__Bacilli; o__Staphylococcales; f__Staphylococcaceae; g__Staphylococcus; s__Staphylococcus_carnosus	0.999948119	66.903 84674	916	TRUE
7	d__Bacteria; p__Firmicutes; c__Bacilli; o__Staphylococcales; f__Staphylococcaceae; g__Staphylococcus; s__Staphylococcus_carnosus	0.999968473	62.209 6049	911	TRUE

8	d__Bacteria; p__Actinobacteriota; c__Actinobacteria; o__Propionibacteriales; f__Propionibacteriaceae; g__Cutibacterium	0.965318867	59.176 61221	863	TRUE
9	d__Bacteria; p__Firmicutes; c__Bacilli; o__Lactobacillales; f__Streptococcaceae; g__Streptococcus; s__Streptococcus_pneumoniae	0.991288542	39.792 08082	901	TRUE
10	d__Bacteria; p__Proteobacteria; c__Gammaproteobacteria	0.994014897	31.602 66521	880	TRUE
11	d__Bacteria; p__Actinobacteriota; c__Actinobacteria; o__Corynebacteriales; f__Nocardiaceae; g__Nocardia; s__Streptomyces_sp.	0.999151643	27.882 96623	872	TRUE
12	d__Bacteria; p__Firmicutes; c__Bacilli; o__Staphylococcales; f__Staphylococcaceae; g__Staphylococcus; s__Staphylococcus_carnosus	0.999981875	27.580 60029	880	TRUE
13	d__Bacteria; p__Actinobacteriota; c__Actinobacteria	0.999956315	19.470 95551	852	TRUE
	COVID Lab				
1	d__Bacteria; p__Actinobacteriota; c__Actinobacteria; o__Corynebacteriales; f__Nocardiaceae; g__Nocardia; s__Streptomyces_sp.	0.999978453	108.69 81382	1194	TRUE
2	d__Bacteria; p__Proteobacteria; c__Gammaproteobacteria; o__Burkholderiales; f__Comamonadaceae; g__Comamonas; s__uncultured_compost	0.994337755	60.074 33825	1188	TRUE
3	d__Bacteria; p__Proteobacteria; c__Alphaproteobacteria	0.99246677	57.124 89899	1179	TRUE
4	d__Bacteria; p__Proteobacteria; c__Alphaproteobacteria; o__Rhizobiales; f__Rhizobiaceae; g__Allorhizobium-Neorhizobium-Pararhizobium-Rhizobium; s__[Pseudomonas]_geniculata	0.99308386	53.615 75452	1187	TRUE
5	d__Bacteria; p__Proteobacteria; c__Alphaproteobacteria; o__Sphingomonadales; f__Sphingomonadaceae; g__Zymomonas; s__Zymomonas_mobilis	0.998143978	52.365 37312	1188	TRUE
6	d__Bacteria	0.999984214	43.287 18119	1142	TRUE
7	d__Bacteria; p__Proteobacteria; c__Gammaproteobacteria	0.996096408	42.682 62463	1185	TRUE
8	d__Bacteria; p__Proteobacteria; c__Gammaproteobacteria	0.997501688	42.165 31706	1180	TRUE
9	d__Bacteria	0.999974292	41.886 27318	1104	TRUE
10	d__Bacteria; p__Firmicutes; c__Bacilli; o__Staphylococcales; f__Staphylococcaceae; g__Staphylococcus; s__Staphylococcus_carnosus	0.999968473	40.538 80097	1167	TRUE
11	d__Bacteria; p__Proteobacteria; c__Alphaproteobacteria	0.999783711	38.423 08988	1144	TRUE

12	d__Bacteria; p__Proteobacteria; c__Alphaproteobacteria; o__Rhizobiales	0.998431465	36.43939099	1128	TRUE
13	d__Bacteria; p__Actinobacteriota; c__Actinobacteria; o__Corynebacteriales; f__Nocardiaceae; g__Nocardia; s__Streptomyces_sp.	0.99996539	26.09147001	1126	TRUE
14	d__Bacteria; p__Firmicutes; c__Bacilli; o__Lactobacillales; f__Streptococcaceae; g__Streptococcus; s__Streptococcus_pneumoniae	0.991288542	21.92085357	1116	TRUE
